# Supplementary material for: Single-Cell RNA Sequencing Reveals a Dynamic Stromal Niche That Supports Tumor Growth
Source: Cell Rep. 2020 May 19;31(7):107628. doi: 10.1016/j.celrep.2020.107628 (PMC7242909; doi:10.1016/j.celrep.2020.107628)
Supplement: Document S1. Figures S1–S7 [file mmc1.pdf]

Cell Reports, Volume *31*

Supplemental Information

Single-Cell RNA Sequencing Reveals a Dynamic Stromal Niche That Supports Tumor Growth

Sarah Davidson, Mirjana Efremova, Angela Riedel, Bidesh Mahata, Jhuma Pramanik, Jani Huuhtanen, Gozde Kar, Roser Vento-Tormo, Tzachi Hagai, Xi Chen, Muzlifah A. Haniffa, Jacqueline D. Shields, and Sarah A. Teichmann

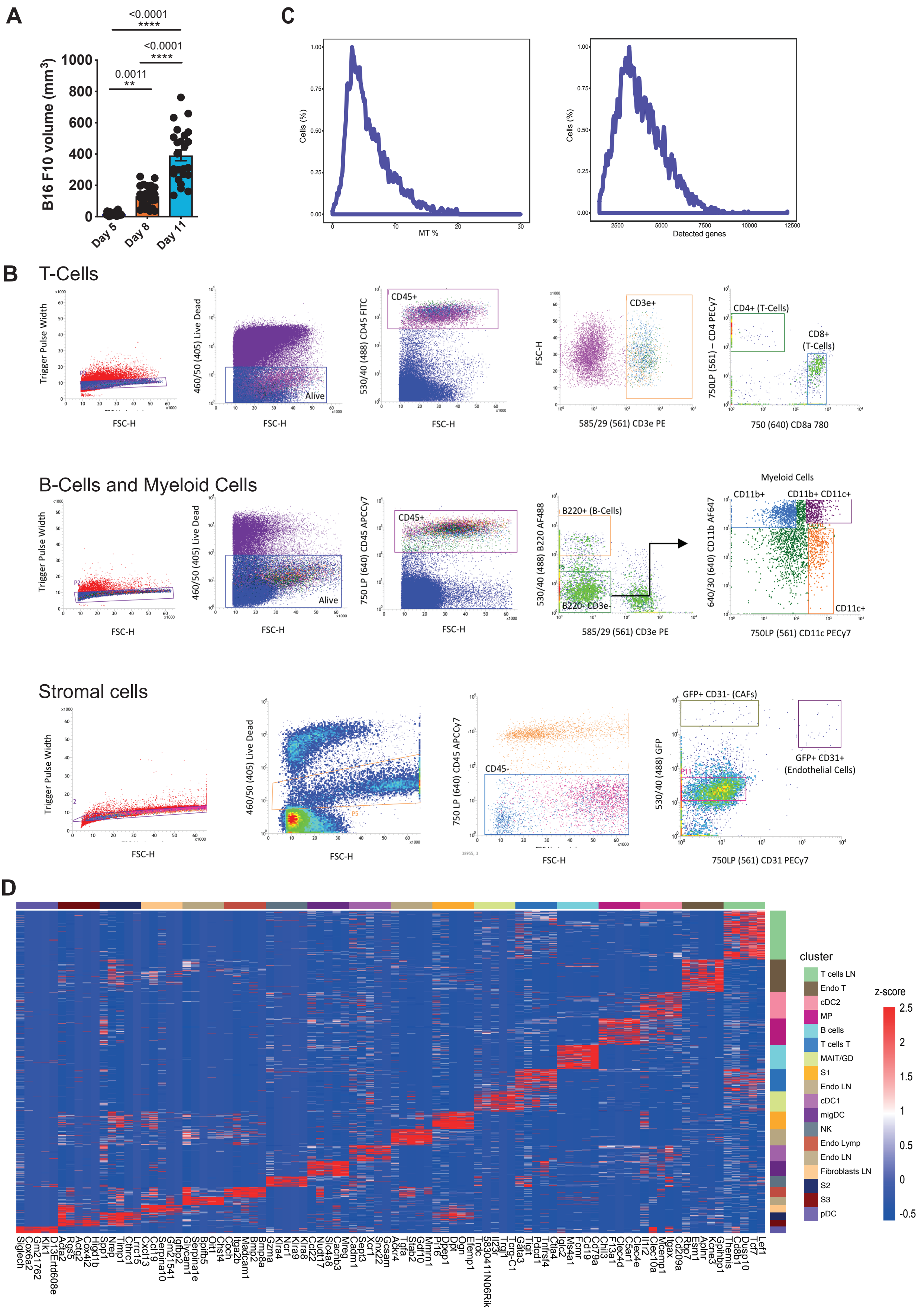

Supplementary Figure 1. Gating strategy and quality control of SS2 data, Related to Figure

1. (A) Volumes of B16-F10 melanomas at day 5, day 8 and day 11. Data presented as mean ± SEM * $P<0.05$, ** $P<0.01$, *** $P<0.001$, **** $P<0.0001$, one way Anova with Tukey post-hoc test, n = 22 individual mice. **(C)** Gating strategy for index sorted cell populations. **(B)** Quality control of the scRNA-seq dataset. Histograms show the distribution of the cells that passed the computational quality control ordered by number of detected genes and mitochondrial gene expression content. **(D)** Heatmap of marker genes showing relative expression (z-score) of the top 5 markers for each cluster presented in Fig. 1B.

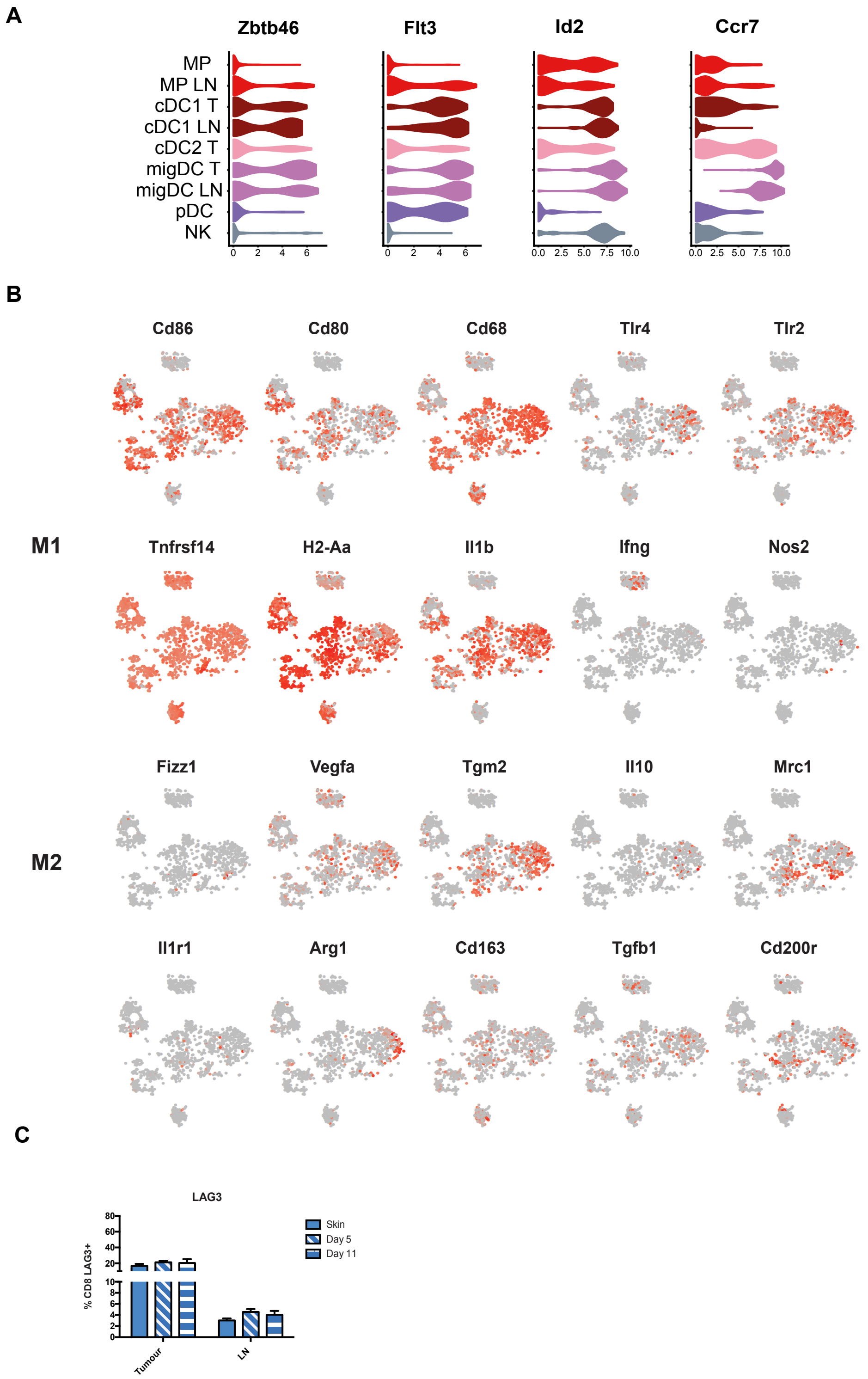

Supplementary Figure 2. Immune phenotypes, Related to Figures 2 and 3. (A) Violin plots showing expression of cDC1 transcription factors for each innate immune subpopulation. **(B)** tSNE plots showing expression of M1 and M2 macrophage markers in the innate immune subpopulations. Red indicates high expression, grey indicates low expression. **(C)** Percentage

of Lag3$^+$ (displayed as a percentage of total CD8$^+$ T cells), in tumours and lymph nodes isolated from skin, day 5 and day 11 tumour bearing or control mice. Data presented as mean ± SEM. n=4 independent mice per time point.

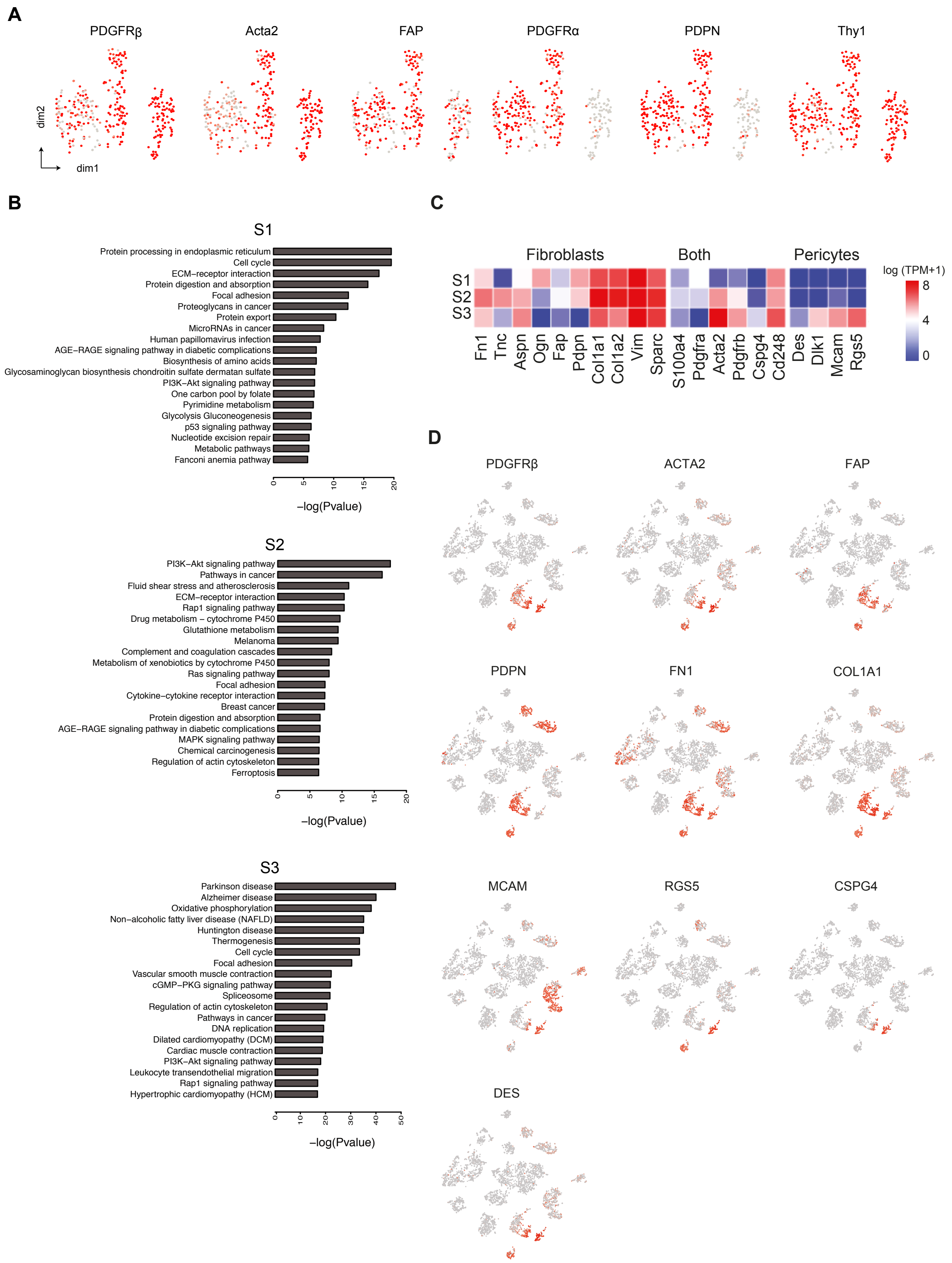

Supplementary Figure 3. Distinct stromal subpopulations identified in the melanoma mouse model, Related to Figure 4. (A) tSNE plots showing expression of typical CAF markers. **(B)** Bar plot (-log pvalue) depicting the top 20 gene ontology pathways upregulated in each stromal population. **(C)** Heatmap showing expression of canonical fibroblasts and pericytes

markers in the three stromal populations. **(D)** tSNE plots of all sequenced cells, showing expression of typical pericyte markers is also detected in $PDPN^+$ lymph node fibroblasts.

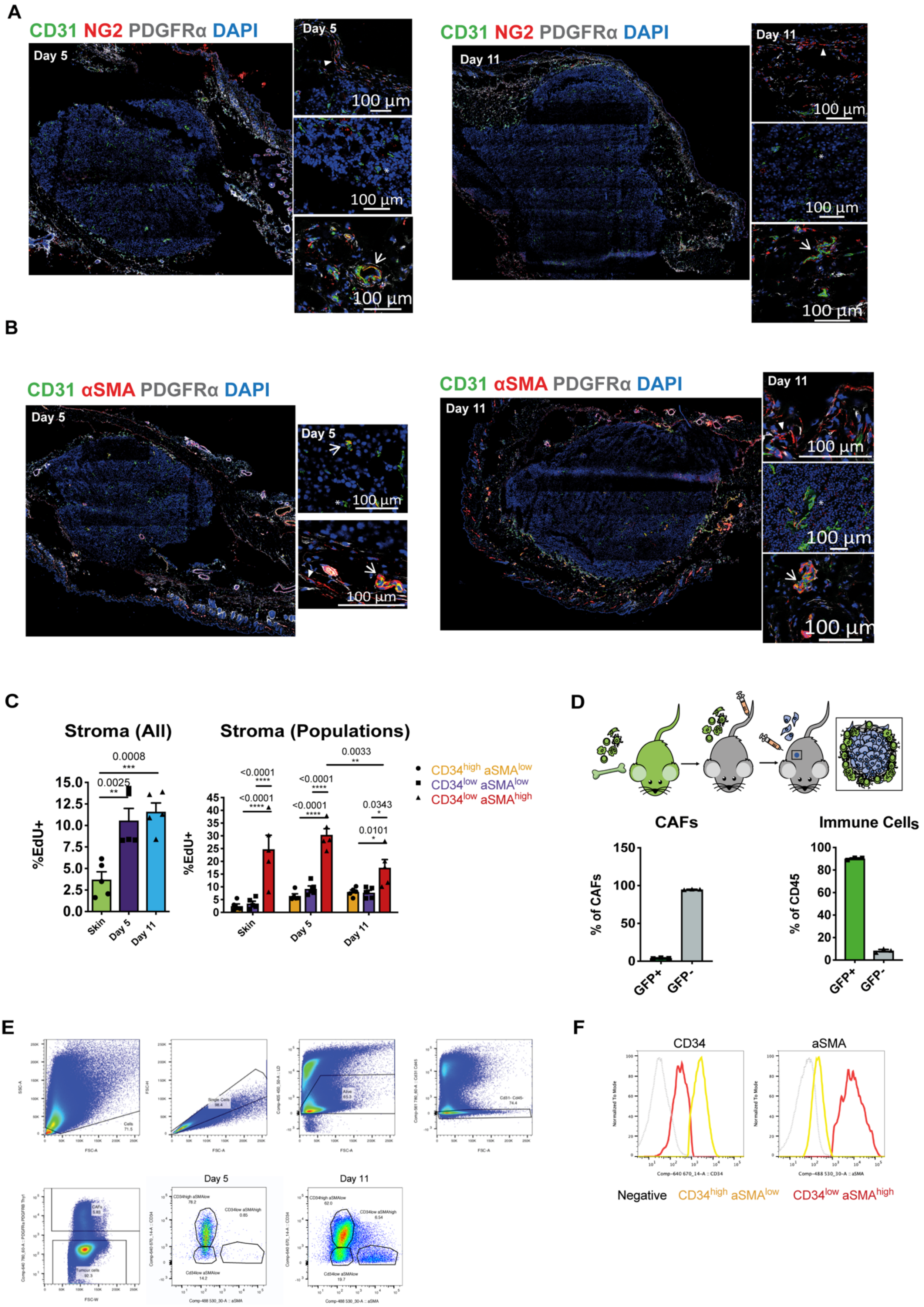
A
CD31 NG2 PDGFRα DAPI
Day 5
100 µm
Day 11
B
CD31 αSMA PDGFRα DAPI
Day 5
Day 11
100 µm
C
Stroma (All)
Stroma (Populations)
%EdU+
Skin
Day 5
Day 11
0.0008
0.0025
0.0033
<0.0001
0.0343
0.0101
CD34high aSMAlow
CD34low aSMAlow
CD34low aSMAhigh
D
CAFs
Immune Cells
% of CAFs
% of CD45
GFP+
GFP-
E
Day 5
Day 11
F
CD34
aSMA
Negative
CD34high aSMAlow
CD34low aSMAhigh

Supplementary Figure 4. Validation of CAF markers and CAF origin, Related to Figures 4 and 5. (A) and **(B)** Confocal imaging showing αSMA^{+} and NG2^{+} cells both distinct from and associated with CD31^{+} blood vessels. 20x tile scans of whole tumours are displayed, as well as 63x regions of interest. **(C)** EdU incorporation in stromal populations in skin, day 5 and day 11 tumours (left: % of CD45- CD31- Thy1+, right; individual subsets (S1-S3) incorporation into all proliferating fibroblasts (top) and each subset (bottom) in skin, day 5 and day 11 tumours. * $P<0.05$, ** $P<0.01$, *** $P<0.001$, **** $P<0.0001$ One way (left) or two way (right) ANOVA with a Tukey post-hoc test. **(D)** Diagram (top) depicting the generation of bone marrow chimeric mice by injecting (IV) bone marrow from CAG-EGFP mice into irradiated WT strains. B16-F10 tumours were injected into chimeric mice and allowed to form for 11 days. The proportion of bone derived marrow cells (GFP+) to stromal (CD45- CD31- Thy1+, bottom left) and immune (CD45+, bottom right) populations was assessed by flow cytometry. **(E)** Gating strategy used to identify stromal subsets along tumour development, **(F)** showing representative plots from day 5 and 11 tumours and histograms of CD34 and aSMA levels between populations. Scale bars 100 µm

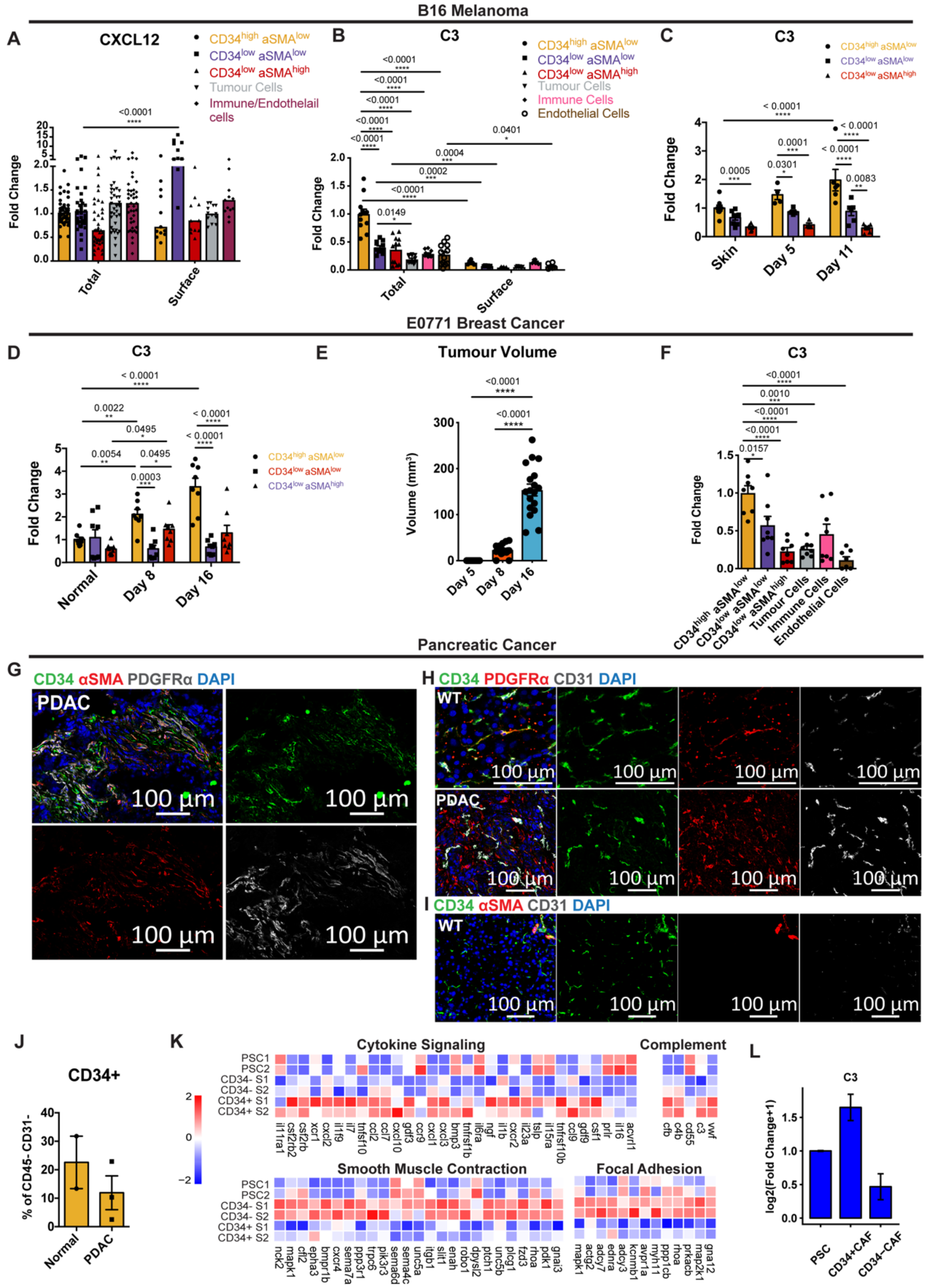

Supplementary Figure 5. Validation of stromal populations in mouse models of melanoma, breast and pancreatic cancer, Related to Figures 5 and 6. B16-F10 melanoma: Total and surface CXCL12 **(A)** and C3 **(B)** expression in day 11 tumours in stromal populations, immune, endothelial and tumour cells. CXCL12: n = minimum of 12 mice, C3: n = 6 mice. **(C)** C3 expression within each stromal population in normal skin, day 5 and day 11 tumours n =

minimum 4 mice. E0771 breast tumours: **(D)** C3 expression within fibroblast subsets in normal breast tissue, day 8 and day 16 tumours, n = 8 mice. **(E)** Tumour volumes at day 5, day 8 and day 16, n = 18 mice. **(F)** C3 expression in fibroblast populations, immune, endothelial and tumour cells in day 16 tumours, n = 8 mice. Pancreatic adenocarcinoma: **(G)** IF images of KPC tumours showing expression of CD34 (green), αSMA (red) and PDGFRα (grey). **(H)** IF images of WT pancreas compared to KPC tumours (PDAC), showing expression of CD34 (green), PDGFRα (red) and CD31 (grey). Pancreatic stellate cells in normal tissue express CD34 and PDGFRα, whereas CD34 expression is mostly restricted to CD31+ blood vessels in PDAC tissue. **(I)** IF images showing CD34 (green), αSMA (red) and CD31 (grey) in normal pancreatic tissue. Here, pancreatic stellate cells do not express αSMA. **(J)** Graph displays FACs quantification of CD34+ fibroblasts as a percentage of CD45-CD31- cells in normal pancreatic tissue and KPC tumours. normal pancreas: n = 2 mice, KPC tumours: n = 3 mice. **(K)** Publicly available RNA seq data of KPC CD34+ and CD34- stromal cells, as well as normal pancreatic stellate cells (PSCs) was downloaded and analysed. Heat maps show selected pathways from GO analysis performed on differentially expressed genes between CD34+ and CD34- populations, z scores are displayed. **(L)** Graph displaying log2(Fold change+1) of C3 RNA expression from PSCs, CD34+ and CD34- stromal cells. Graphs were made from previously mentioned publicly available data. ** $P<0.01$, *** $P<0.001$, **** $P<0.0001$, two way anova with a Sidak (A and B) and Tukey (C and D) post-hoc test, one way anova with Tukey post-hoc test. (E and F). All images are representative of at least n=3 mice and acquired at 63x, scale bars displayed.

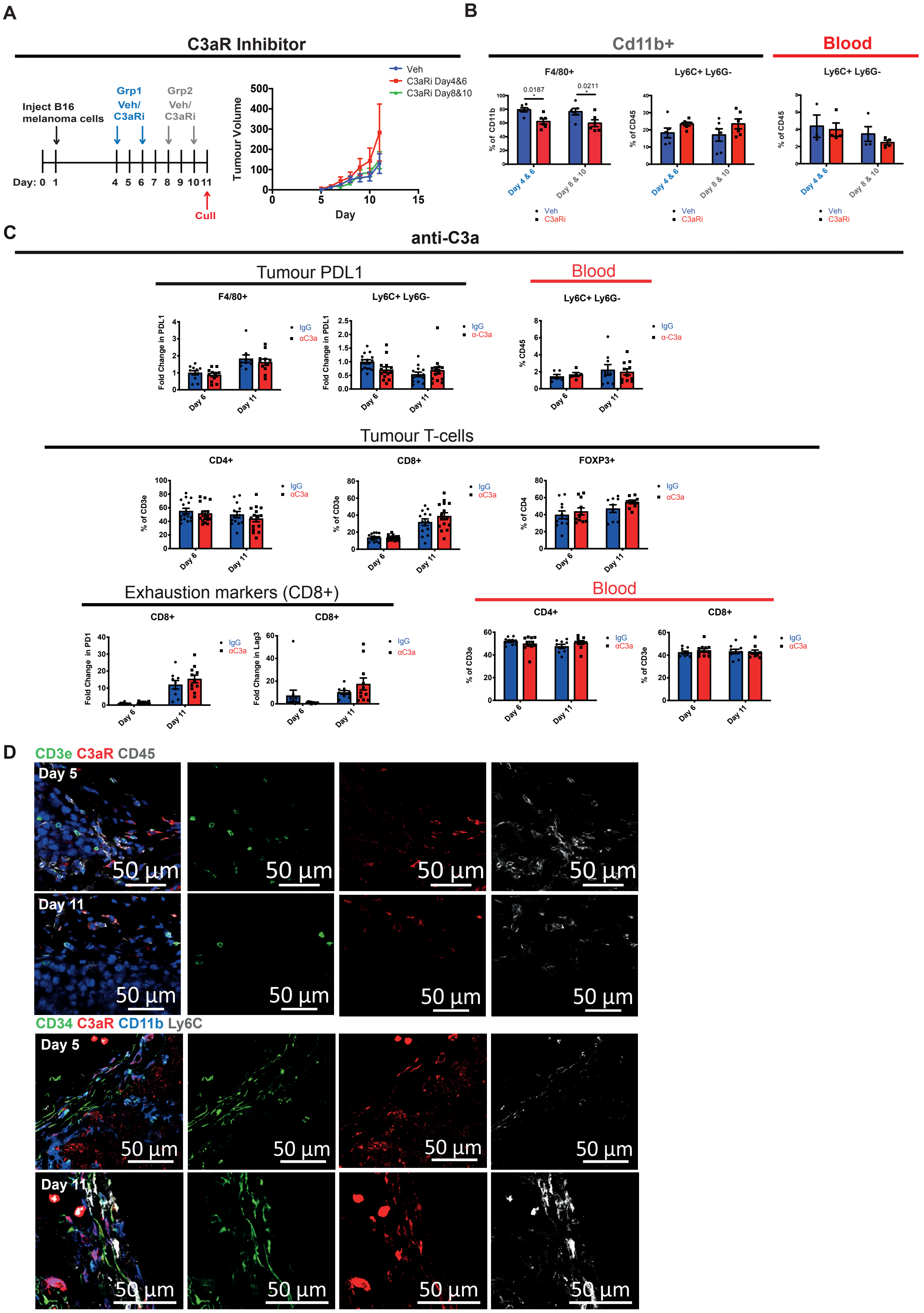
A
C3aR Inhibitor
Inject B16 melanoma cells
Grp1 Veh/ C3aRi
Grp2 Veh/ C3aRi
Day: 0 1 4 5 6 7 8 9 10 11
Cull
Tumour Volume
Day
Veh
C3aRi Day4&6
C3aRi Day8&10
B
Cd11b+
Blood
F4/80+
Ly6C+ Ly6G-
Ly6C+ Ly6G-
0.0187
0.0211
% of CD11b
% of CD45
% of CD45
Day 4 & 6
Day 8 & 10
Veh
C3aRi
C
anti-C3a
Tumour PDL1
Blood
F4/80+
Ly6C+ Ly6G-
Ly6C+ Ly6G-
Fold Change in PDL1
% CD45
IgG
αC3a
α-C3a
Day 6
Day 11
Tumour T-cells
CD4+
CD8+
FOXP3+
% of CD3e
% of CD4
Exhaustion markers (CD8+)
CD8+
CD8+
Fold Change in PD1
Fold Change in Lag3
Blood
CD4+
CD8+
% of CD3e
D
CD3e C3aR CD45
Day 5
Day 11
50 µm
CD34 C3aR CD11b Ly6C
Day 5
Day 11
50 µm

Supplementary Figure 6. The effects of disrupting C3a/C3aR Interactions on tumour development and immune infiltration, Related to Figure 6. (A) Experimental design of C3aR antagonism, using the small molecule SB290157: Mice received injections of SB290157 at either day 4 and day 6 or day 8 and day 11, all mice were culled at day 11 (left). Tumour volumes (mm^3) for vehicle controls and each treatment regime are shown (right), n = 6 mice. **(B)** Immune infiltration of F4/80+ macrophages and Ly6C+ Ly6G- monocytes in the tumour (left) and blood (right). This is shown as the percentage of CD11b+ cells (F4/80) or CD45+ cells (Ly6C+ Ly6G-), n = 6 tumours. * = P< 0.05 (Two way anova with Sidak post-hoc test). **(C)** The effects of neutralising C3a on immune infiltrates in the tumour and blood. Top panel: Fold change in fluorescence intensity of PDL1 on tumour myeloid populations, normalized to IgG day6 (left) and the number of Ly6C+ Ly6G- monocytes in the blood, shown as a percentage of CD45 cells. Middle panel: Number of tumour infiltrating CD8+, CD4+ and FOXP3+ T-cells, displayed as a percentage of CD3e or CD4 T-cells. Bottom panel: Fold change in fluorescence intensity of T-cell exhaustion markers PD1 and Lag3 in CD8 T cells, normalized to IgG day 6 (left). Numbers of CD8 and CD4 T cells in the blood of treated mice, displayed as a percentage of CD3e T-cells. n = minimum of 11 mice. **(D)** Confocal images showing expression of C3aR on immune populations in day 5 and 11 tumours. Top panel: C3aR is not expressed on Cd3e+ T cells, CD3e (green), C3aR (red) and CD45 (grey). Bottom panel: C3aR is expressed by Ly6C+ CD11b+ monocytes. Images were acquired at 63x and scale bars are displayed. day 5 n=3 tumours, day 11 n=2 tumours.

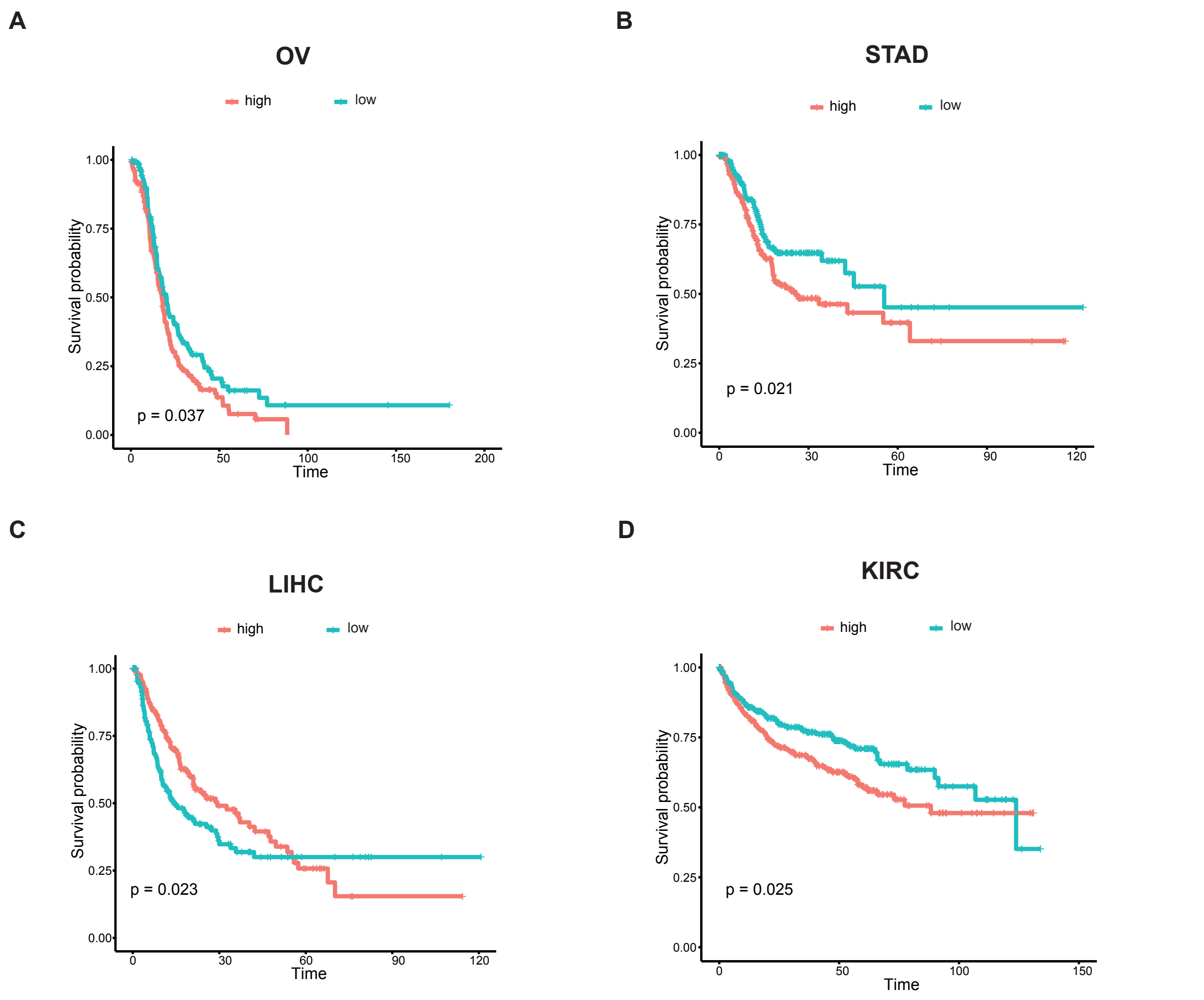

Supplementary Figure 7. Survival analysis of C3 in different TCGA datasets, Related to Figure 7. (A) Kaplan-Meier survival curve of Ovarian serous cystadenocarcinoma (OV) patients showing progression-free survival based on median C3 expression levels. **(B)** Kaplan-Meier survival curve of Stomach adenocarcinoma (STAD) patients showing progression-free survival based on median C3 expression levels. **(C)** Kaplan-Meier survival curve of Liver hepatocellular carcinoma (LIHC) patients showing progression-free survival based on median C3 expression levels. **(D)** Kaplan-Meier survival curve of Kidney renal clear cell carcinoma (KIRC) patients showing progression-free survival based on median C3 expression levels.
