# Supplementary material for: Single-Cell RNA Sequencing Reveals a Dynamic Stromal Niche That Supports Tumor Growth
Source: Cell Rep. 2020 May 19;31(7):107628. doi: 10.1016/j.celrep.2020.107628 (PMC7242909; doi:10.1016/j.celrep.2020.107628)
Supplement: Document S2. Article plus Supplemental Information [file mmc6.pdf]

Cell Reports

Article

Single-Cell RNA Sequencing Reveals a Dynamic Stromal Niche That Supports Tumor Growth

Graphical Abstract

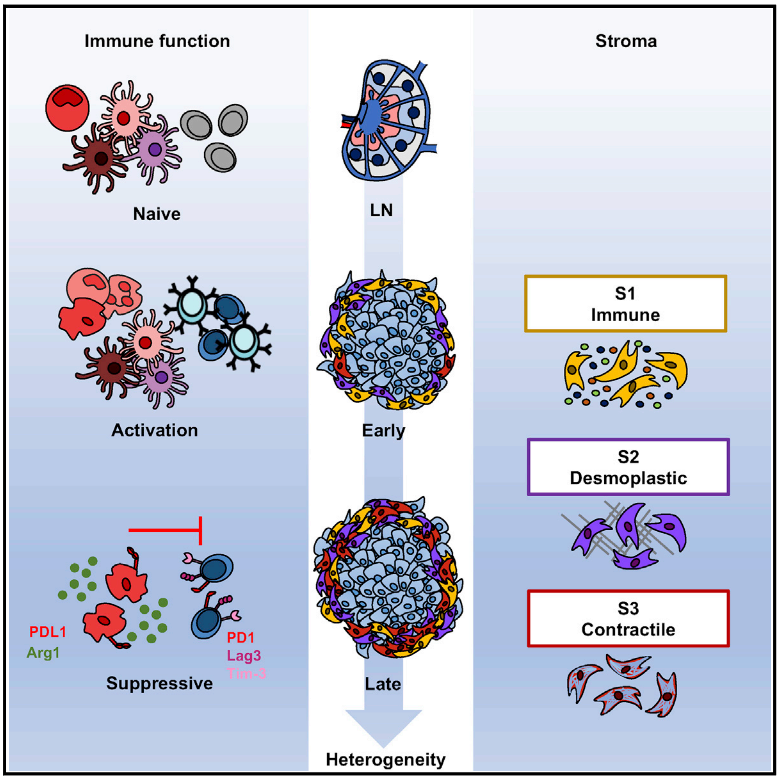

Highlights

- scRNA-seq reveals the complex interplay among cells within the evolving tumor
- T cells recruited from lymph nodes are activated and clonally expand *in situ*
- Temporally regulated, functionally distinct stromal populations exist
- Cross-compartment interactions can be identified using the CellPhoneDB database

Authors

Sarah Davidson, Mirjana Efremova, Angela Riedel, ..., Muzlifah A. Haniffa, Jacqueline D. Shields, Sarah A. Teichmann

Correspondence

js970@mrc-cu.cam.ac.uk (J.D.S.),
st9@sanger.ac.uk (S.A.T.)

In Brief

Davidson et al. use scRNA-seq to demonstrate the increasing heterogeneity within the stroma as a tumor develops. They show that T cell activation occurs at the tumor following recruitment from lymph nodes and reveal the presence of three dynamic stromal populations that display unique functional properties.

Davidson et al., 2020, Cell Reports *31*, 107628
May 19, 2020 © 2020 The Authors.
https://doi.org/10.1016/j.celrep.2020.107628

CellPress

Cell Reports

CellPress
OPEN ACCESS

Article

Single-Cell RNA Sequencing Reveals a Dynamic Stromal Niche That Supports Tumor Growth

Sarah Davidson,[2,8] Mirjana Efremova,[1,8] Angela Riedel,[2] Bidesh Mahata,[1,6] Jhuma Pramanik,[1] Jani Huuhtanen,[3] Gozde Kar,[1] Roser Vento-Tormo,[1] Tzachi Hagai,[4] Xi Chen,[1] Muzlifah A. Haniffa,[5] Jacqueline D. Shields,[2,8,9,*] and Sarah A. Teichmann[1,7,8,*]

[1]Wellcome Sanger Institute, Wellcome Genome Campus, Hinxton, Cambridge CB10 1SA, UK
[2]Medical Research Council Cancer Unit, University of Cambridge, Hutchison/Medical Research Council Research Centre, Box 197 Cambridge Biomedical Campus, Cambridge, CB2 0XZ, UK
[3]Hematology Research Unit Helsinki, Department of Clinical Chemistry and Hematology, University of Helsinki and Helsinki University Hospital Comprehensive Cancer Center, Helsinki, Finland
[4]School of Molecular Cell Biology and Biotechnology, George S. Wise Faculty of Life Sciences, Tel Aviv University, Tel Aviv 69978, Israel
[5]Institute of Cellular Medicine, Newcastle University, Newcastle upon Tyne NE2 4HH, UK
[6]Department of Pathology, University of Cambridge, Cambridge, UK
[7]Cavendish Laboratory, University of Cambridge, JJ Thomson Ave, Cambridge CB3 0HE, UK
[8]These authors contributed equally
[9]Lead Contact
*Correspondence: js970@mrc-cu.cam.ac.uk (J.D.S.), st9@sanger.ac.uk (S.A.T.)
https://doi.org/10.1016/j.celrep.2020.107628

SUMMARY

Here, using single-cell RNA sequencing, we examine the stromal compartment in murine melanoma and draining lymph nodes (LNs) at points across tumor development, providing data at http://www.teichlab.org/data/. Naive lymphocytes from LNs undergo activation and clonal expansion within the tumor, before PD1 and Lag3 expression, while tumor-associated myeloid cells promote the formation of a suppressive niche. We identify three temporally distinct stromal populations displaying unique functional signatures, conserved across mouse and human tumors. Whereas "immune" stromal cells are observed in early tumors, "contractile" cells become more prevalent at later time points. Complement component C3 is specifically expressed in the immune population. Its cleavage product C3a supports the recruitment of C3aR$^+$ macrophages, and perturbation of C3a and C3aR disrupts immune infiltration, slowing tumor growth. Our results highlight the power of scRNA-seq to identify complex interplays and increase stromal diversity as a tumor develops, revealing that stromal cells acquire the capacity to modulate immune landscapes from early disease.

INTRODUCTION

To aid their growth and development, malignant cells cultivate a supporting niche of "normal" cells, known as the tumor microenvironment (TME). This niche comprises non-immune cells such as fibroblasts, blood and lymphatic endothelial cells, and numerous immune populations (Turley et al., 2015). The balance of anti-tumor versus pro-tumor leukocytes can dictate tumor fate (Galon et al., 2006; Sato et al., 2005), and suppressive populations can persist to support immune escape and prevent tumor clearance. While immunotherapies such as anti-CTLA4, anti-PD1, and anti-PD-L1 show efficacy in a large number of melanoma patients, a significant proportion do not respond to this treatment (Brahmer et al., 2012; Hamid et al., 2013; Hodi et al., 2010; Topalian et al., 2012). Thus, there remains an unmet need to uncover therapeutic targets. The numerous mechanisms through which stromal fibroblasts and immune cells promote tumor growth represent a wealth of opportunities for therapeutic intervention. However, the evolving TME is extremely dynamic, continually adapting to both soluble and mechanical cues, inducing significant heterogeneity within the stromal compartment (Junttila and de Sauvage, 2013).

Cancer-associated fibroblasts (CAFs) are the most abundant stromal component, secreting growth factors, promoting angiogenesis, facilitating metastasis, and regulating immune infiltration (Calon et al., 2012; Dumont et al., 2013; Gaggioli et al., 2007; Guo et al., 2008; Harper and Sainson, 2014; Jia et al., 2013; Orimo et al., 2005). Although they express typical fibroblast markers such as fibroblast activation protein (FAP), platelet-derived growth factor receptors α (PDGFRα) and β (PDGFRβ), podoplanin (PDPN), Thy-1, and α-smooth muscle actin (αSMA), no single marker universally identifies all CAFs within the tumor stroma (Augsten, 2014; Cortez et al., 2014; Roswall and Pietras, 2012). Such barriers to the identification of CAFs in the TME may underpin conflicting evidence for both pro- and anti-tumor activities, which may reflect the existence of subpopulations of cells possessing different functional properties (Feig et al., 2013; Özdemir et al., 2015). Recently, single-

© 2020 The Authors.
This is an open access article under the CC BY license (http://creativecommons.org/licenses/by/4.0/).

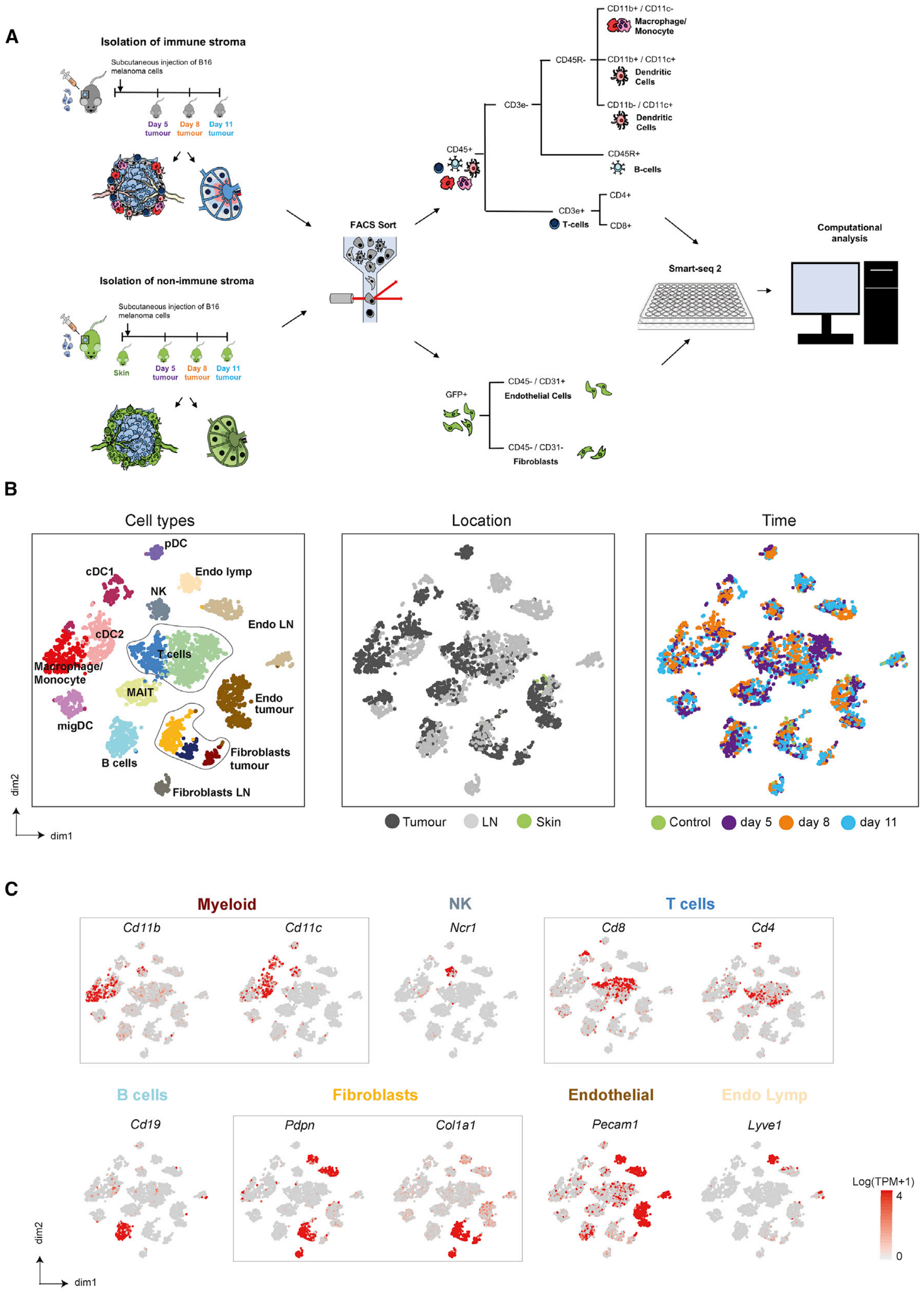

(legend on next page)

cell technologies have yielded insights into the diversity of the TME, and are beginning to reveal the extent of heterogeneity within the stromal compartment (Bartoschek et al., 2018; Costa et al., 2018; Elyada et al., 2019; Lambrechts et al., 2018; Öhlund et al., 2017; Puram et al., 2017). While commonalities between stromal populations were identified across the cancer types examined, differences in functional signatures and marker profiles were observed between tumor types and anatomical location, indicating the existence of site-specific programs. Whether similarly diverse stromal subsets are present within melanoma and how the composition and functions adapt as a tumor develops remain to be explored. Therefore, we used single-cell RNA sequencing (scRNA-seq) to interrogate the developing TME in real time, revealing previously unrecognized traits and an increasing heterogeneity.

Here, we identified the presence of a diverse immune landscape, in which effector T cells displayed signs of dysfunction predominantly in late stages, while myeloid cells concomitantly increased the expression of suppressive molecules. This work also highlighted significant heterogeneity within the stromal compartment of the primary tumor. Three distinct mesenchymal populations were identified—immune, desmoplastic, and contractile—each displaying unique functional and temporal characteristics key to the tumor. At early time points, the immune and desmoplastic populations dominated, yet at later stages, the third contractile subset became more prevalent. Using a unique database of known ligand-receptor interactions, we investigated communication between different stromal and immune populations to reveal the complex interplay between the immune stromal subset, macrophages, and T cells, which ultimately contributes to T cell dysfunction.

RESULTS

Identification of Immune and Stromal Populations within the Developing TME

To reconstruct the immune composition of a developing TME, we injected B16-F10 melanoma cells into mice. At different time points (days 5, 8, and 11) during tumor development, specific immune populations were enriched based on surface marker expression and index sorted from both tumors and lymph nodes (LNs). In this model, early day 5 tumors presented as barely palpable masses compared with late tumors at day 11 (Figure S1A). To avoid the biases associated with the isolation of stromal cells, we also injected B16-F10 melanoma cells into CAG-EGFP mice, which exhibit widespread EGFP expression. This enabled a negative selection approach, which did not rely upon the expression of surface markers. Tumor and immune cells were removed by selecting GFP^{+} CD45^{-} cells only, with the remaining stromal cells separated into CD31^{+} blood and lymphatic endothelial cells and CD31^{-} stromal populations. Single cells were isolated from two animals per time point and profiled using Smart-seq2 (Figures 1A and S1B).

After quality control (see Method Details and Figure S1C), >4,600 cells were sequenced. Using graph-based clustering (Satija et al., 2015) and known marker expression, numerous immune and stromal populations were identified (Figures 1B, 1C, S1D). Clusters denoting T cells, dendritic cells (DCs), and endothelial cells separated according to their location in either the tumor or LN, while other cell types clustered together irrespective of their site of origin. This indicates that particular populations possess site-specific transcriptional programs (Figure 1B). Furthermore, sampling multiple time points across each site enabled us to investigate temporal adaptations within each population (Figure 1B).

We provide these data in a browsable format online at http://www.teichlab.org/data/.

Dynamics of Immune Cells

The innate immune system has the ability to detect malignant cells and coordinate an anti-tumor response. Thus, we sought to investigate relationships within these populations in both the primary tumor and draining LN. Clusters corresponding to natural killer (NK) cells, plasmacytoid DCs (pDCs), and conventional DCs (cDCs), as well as a mixture of macrophages, monocytes, and neutrophils, were identified based on known markers. These included Macrophages/Monocytes/Neutrophils, *Itgam* (Cd11b); *Adgre1*(F4/80); *Fcgr1*; *Ly6c*; *Ly6g*, NK *Ncr1*; pDCs, *Bst2*, *Siglech*; and cDCs, *Itgax* (*Cd11c*) (Figures 2A–2C; Table S1). Moreover, multiple DC populations were observed that reflect the conventional DC (cDC) subsets cDC1 and cDC2. cDC1 and cDC2 titles were assigned based on the expression of known markers, including *Cd11c*, *Clec9a*, *Baft3* (cDC1), *Cd11b*, *Fcgr1*, and *Sirpa* (cDC2) (Figures 2B and 2C). Two further clusters were identified that lacked lineage markers for adaptive immune cells and the classical DC integrins Cd11b and Cd11c, yet expressed DC transcription factors *Zbtb46*, *Baft3*, *Flt3*, and *Id2.* These populations were called migratory DCs (migDCs) owing to the high levels of *Ccr7*; however, they may represent DC precursors that later develop expression of *Cd11c* (Figure S2A).

Each DC population further separated according to their location in either the tumor or draining LN (Figure 2A). cDC1 cells in the tumor expressed the dermal marker *Cd103* (*Itgae*), whereas their LN counterparts expressed *Cd8a*, a marker of LN resident populations, indicating that these cells did not migrate from the tumor. *Cd11b*$^{+}$ mononuclear phagocytes (MPs) in the LN consisted of *Adgre1*$^{+}$ (F4/80) macrophages and *Ccr2*$^{+}$ monocytes,

Figure 1. Distinction of Melanoma Stromal Populations with Single-Cell RNA-Seq

(A) Overview of experimental and sequencing workflow.

(B) t-Distributed Stochastic Neighbor Embedding (tSNE) visualization of all cells sequenced with each cell color coded for cell type (left), site of origin (center), and time (right).

(C) Expression of marker genes for each cell type. n = 32 mice.

cDC1/2, conventional dendritic cell; DC LN, lymph node dendritic cell; Endo lymph, lymphatic endothelial cell; endo LN, lymph node endothelium; Endo tumor, tumor endothelial cells; fibroblast LN, lymph node fibroblast; MAIT, mucosal-associated invariant T cell; migDC, migratory DC; NK, natural killer; pDC, plasmacytoid DC.

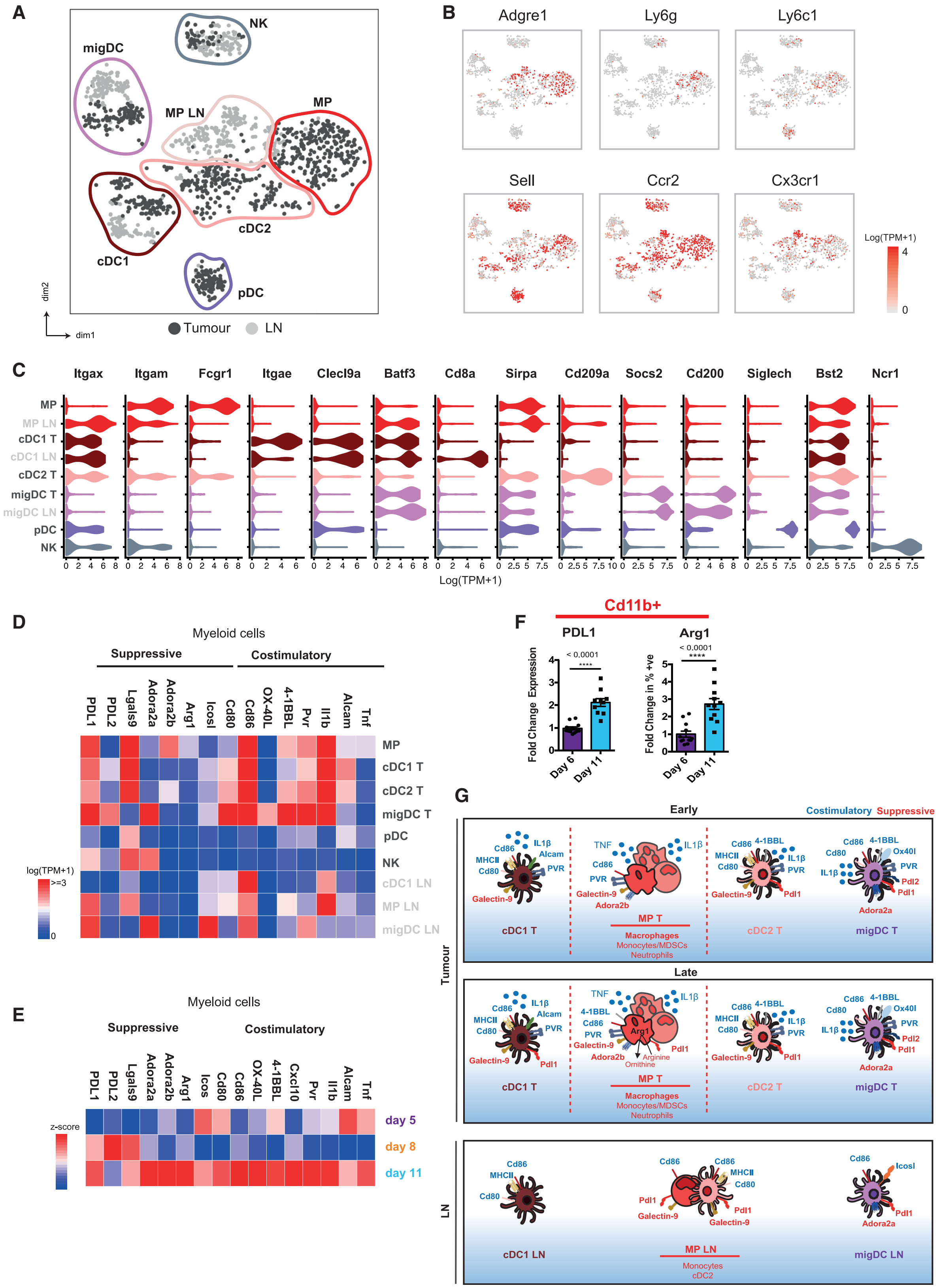

(legend on next page)

as well as a resident *Cd11c*$^{+}$ cDC2 population (Figure 2B). A comparison of equivalent tumor and LN clusters revealed that myeloid cells located in the tumor displayed a more activated phenotype. Tumor resident cells showed increased expression of co-stimulatory molecules *Alcam*, *Pvr*, *Tnfsf9* (4-1BBL), and *Tnfsf4* (OX-40L) and inflammatory cytokines *Il1β* and *Tnf*. However, tumor macrophages, cDC1 cells, and migDCs were also more immunosuppressive, displaying higher levels of *Arg1 (*arginase-1), *Lgals9* (galectin-9), *Cd247* (Pdl1), and *Pdcd1lg2* (Pdl2), respectively (Figure 2D). Although tumor macrophages expressed suppressive markers, no clear delineation between an M1 or the pro-tumor M2 phenotype was observed (Figure S2B). Within the tumor, expression of immunosuppressive molecules, including *Cd274* (PDL1) and *Arg1*, increased at later time points. This temporal change in expression was further confirmed at the protein level, within tumor Cd11b^{+} cells (Figures 2E and 2F). This indicates that tumor resident myeloid populations are present and activated at early stages of tumor growth, yet become more suppressive as the tumor progresses (Figure 2G). However, this phenomenon does not extend to the draining LN, suggesting a subdued inflammatory response at this site. This is particularly relevant in regard to cDC1 cells, which can cross-present tumor antigen to cytotoxic T lymphocytes.

T cell populations from tumors and draining LNs were also transcriptionally distinct, clustering based upon their subtype and location (Figure 3A). At the LN, T cells exhibited a more naive phenotype compared to those present at the tumor (Figure 3B). While tumor resident CD4^{+} T cells were more activated, a significant proportion highly expressed Treg-associated genes at the tumor (Figure 3B). Similarly, within the CD8^{+} T cell compartment, those at the tumor were also more activated, expressing high levels of *Ifng* (interferon γ [IFNγ]), *Prf1* (perforin), and *Gzmb* (granzyme B). However, these cells were also less functional, which is evident in the expression of *Pdcd1* (pd1*)*, *Lag3*, and *Tim3* (Figure 3B). To identify transcriptional adaptations in CD8^{+} T cells at the different stages of tumor development, we performed a pseudotime analysis that revealed a trajectory of gene expression associated with functional changes in these cells. This confirmed that the majority of T cells within the lymph node were naive, displaying high expression of *Sell* and *Tcf7* (Figures 3C and 3D; Table S2). Arrival at the tumor corresponded with the acquisition of activation signatures, including the upregulation of *Ifng* and *Gzmb*. Furthermore, T cell receptor sequencing analysis identified clonal expansion (Figures 3C), specifically within tumors at later time points. This was accompanied by the expression of proliferation marker *Mki67* and exhaustion markers *Pdcd1*, *Lag3*, and *Tim3* at the RNA level (Figures 3C and 3D), which is consistent with reports of cell differentiation from naive cells, through a transitional state, toward dysfunction in human melanoma (Li et al., 2019). Furthermore, a highly proliferative, early dysfunctional population, consistent with our proliferative exhausted population, was also observed in the same study (Li et al., 2019). Flow cytometry analysis confirmed enhanced tumor-infiltrating CD8^{+} T cells with concurrent tumor-specific proliferation and increasing PD1 expression, at later time points (Figures 3E). A tumor-specific increase in Lag3 expression compared to LNs was also detected at the protein level (Figure S2C). A subset of the potentially exhausted CD8^{+} T cells also showed the expression of Entpd1 (CD39), which was recently identified as a marker to distinguish tumor-specific and bystander CD8^{+} T cells (Simoni et al., 2018). These results indicate that T cell recruitment from the LN is followed by activation and subsequent functional defects *in situ*. These functional defects correspond with the gain of immunosuppressive properties in myeloid populations at later time points, indicating that the immune stroma transitions from immunogenic to suppressive phenotypes.

Tumor Stroma Comprise Three Distinct Functional Populations

As the stroma is emerging as a potent immune modulator, we also examined this compartment during tumor progression. We identified three distinct CD31^{-} stromal populations, referred to as Stromal 1, 2, and 3 (Figure 4A; Table S3). The expression of commonly used mesenchymal markers confirmed this identity, but as expected, individual markers were extremely variable from cell to cell and across clusters (Figures 4B and S3A). However, marker combinations could be associated with particular clusters. Stromal 1(S1) was distinguished from S3 by high levels of *Pdpn, Pdgfrα*, and *Cd34*, while *Acta2* (αSMA) was strongly expressed by the latter. S2 represented an intermediate population that expressed *Pdpn* and *Pdgfrα*, yet displayed low expression of *Acta2* and *Cd34* (Figure 4B).

Each cluster displayed distinct functional signatures (Figures 4C and S3B), indicating the existence of specific roles within the TME. S1 (*Pdpn^{high} Pdgfra^{high} Cd34^{high}*) likely engages in immune crosstalk; upregulating genes involved the recruitment and regulation of immune cells. These included the cytokines *Cxcl12*, *Csf1*, and *Ccl8*; cytokine receptors *Il6ra* and *Il6st*; and components of the complement cascade *C3*, *C2*, *and C4b*. In contrast, S2 (*Pdpn^{high} Pdgfra^{high} Cd34^{low}*) upregulated genes encoding extracellular matrix (ECM) components, including numerous collagen family members such as *Postn* and *Tnc.* These ECM components are strongly associated with a fibrotic

Figure 2. Myeloid Cell Clusters in the Tumor Exhibit Suppressive Characteristics

(A) tSNE plot of individual myeloid cells colored by site (tumor, dark gray; lymph node, light gray) and clusters marked by colored lines.

(B) tSNE plots showing the expression of selected marker genes for macrophages and inflammatory and resident monocytes.

(C) Violin plots showing the expression of selected surface marker genes within each cell cluster displayed as log (TPM+1). TPM, transcript count per million.

(D) Heatmap showing mean expression (log(TPM+1)) of co-stimulatory and suppressive genes for the identified cell clusters.

(E) Heatmap showing the relative expression (*Z* score) of co-stimulatory and suppressive genes in all innate immune cells over time.

(F) Flow cytometric analysis of tumor infiltrating CD11b^{+} cells for the expression of suppressive markers PDL1 and Arg 1 at days 6 and 11. Data presented as means ± SEMs; day 6 n = 12 independent mice and day 11 n = 11 independent mice. ****$p < 0.0001$ (t test).

(G) Schematic diagram of the co-stimulatory and inhibitory receptors-ligands expressed on distinct myeloid subpopulations.

For (A)–(E) and (G), n = 17 mice. cDC1/2, conventional dendritic cell; pDC, DC LN, lymph node dendritic cell; migDC, migratory DC; MP, mononuclear phagocyte; plasmacytoid DC.

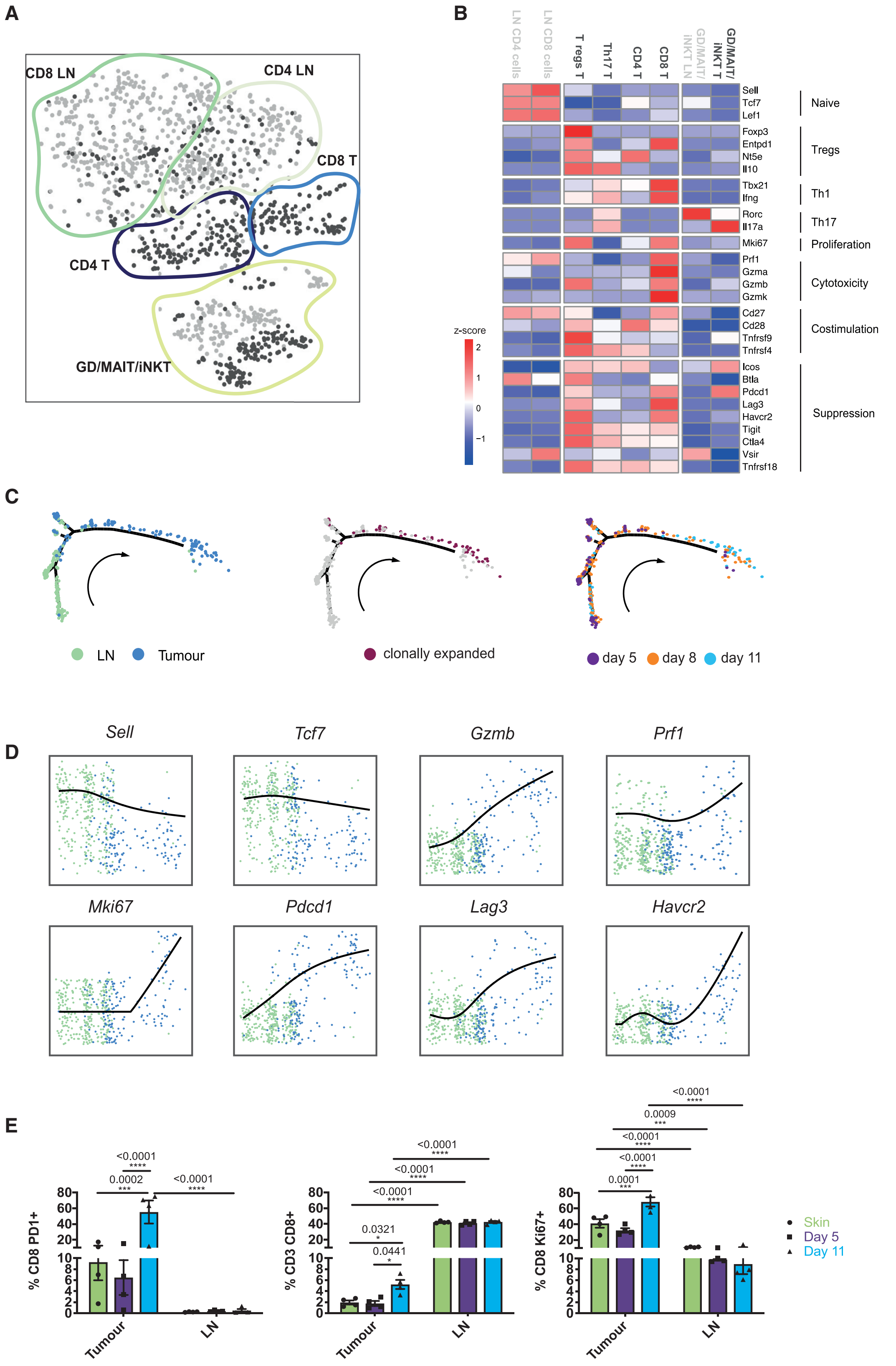

(legend on next page)

CellPress
OPEN ACCESS

matrix, a feature common to developed tumors (Bonnans et al., 2014) and implicated in immune exclusion. Thus, this population may drive the desmoplastic reaction. S3 (*Acta2*^{high}) likely represents a more contractile stromal subset, expressing genes involved in the regulation and rearrangement of the actin cytoskeleton. In particular, this cluster upregulated *Rock1*, *Mlc2*, and *Mlck*, which are responsible for the contraction of actin stress fibers. While all three stromal populations expressed fibroblast markers, indicating that they represent melanoma CAFs, S3 also expressed some pericyte-associated markers such as *Cspg4* (Ng2), *Mcam*, and *Rgs5* (Figure S3C). Many of the same markers were detected in LN *Pdpn*$^{+}$ fibroblasts (FRCs) (Figure S3D), indicating promiscuous expression that is not limited to pericytes. Thus, to clarify whether S3 represents a pericyte or fibroblast population in murine melanoma, we examined the expression of αSMA and neuron-glial antigen 2 (NG2) in relation to the endothelial marker CD31. While NG2^{+} and αSMA^{+} cells were observed surrounding vessels in adjacent skin, they were less frequently associated with intratumoral vessels. Furthermore, both markers could be detected in peritumoral spindle-shaped cells that are distinct from the vasculature (Figures S4A and S4B). Thus, the precise cellular identity of S3 remains elusive and may represent both pericytes associated with the vasculature and more fibroblast-like cells that are dissociated from vessels.

The collection of samples at different time points across tumor growth enabled us to examine the dynamics of the stromal compartment as a tumor develops. Each population was detected throughout the time course; however, clusters dominated at different points. The stromal compartment from early day 5 tumors primarily comprised S1 and S2, whereas the S3 population was largely restricted to later stages, implying a selective enrichment in established tumors (Figures 4D and 4E). While cells within S1 resembled tissue resident fibroblasts of both mouse and human skin (Figure 4G), *Mki67* was observed specifically in S2 and S3, supporting the concept of proliferative enrichment at later time points (Figure 4F). Increasing proliferation in S3 was confirmed by the incorporation of the thymidine analog EdU (Figure S4C). Recruitment of bone marrow-derived mesenchymal cells to the TME has also been reported (Direkze et al., 2004; Quante et al., 2011; Raz et al., 2018). To investigate whether this alternate source contributes to the expansion of S3, bone marrow chimeric mice were generated (Figure S4D). In our hands, few bone marrow-derived stromal cells (GFP^{+}) were detected, suggesting a negligible influence on the tumor stromal niche in this model.

We next used the marker repertoires identified to validate these different populations in the TME. Consistent with our sequencing data, confocal imaging revealed that the S1/S2 markers PDPN and PDGFRα largely colocalized, while the expression of αSMA was more distinct (Figure 5A). The immune S1 marker CD34, colocalized with both PDPN and PDGFRα, indicated the presence of a CD34^{high}αSMA^{low} stromal subset (Figure 5A). However, some colocalization between αSMA and PDGFRα, PDPN, and CD34 was observed. This may represent the intermediate PDPN^{high} PDGFRα^{high} S2 population, which also expressed low levels of CD34 and αSMA. Flow cytometry further confirmed the presence of CD34^{high} αSMA^{low} (S1), CD34^{low} αSMA^{low} (S2) in normal skin, and within tumors (Figures 5B, S4E, and S4F). In contrast, CD34^{low} αSMA^{high} (S3) was rare in normal skin, becoming most prevalent at later time points (Figures 5B, S4E, and S4F), in line with kinetics described in sequencing data. To explore the inflammatory phenotype associated with S1 in more detail, we focused on the immunomodulatory factors CXCL12 and CSF1 and the complement component C3. At the protein level, intracellular CXCL12 expression was higher in S1 and S2 than in S3 (Figure 5C). While S1 produced CXCL12, high levels were also detected in S2; however, the majority of this was surface associated, which is indicative of extracellular binding specifically to this population (Figure S5A). In contrast, complement component C3 was consistently and predominantly detected in S1 cells across all of the time points examined (Figures 5C, S5B, and S5C). Confocal imaging also showed CD34^{high} CAFs to be a source of CSF1 in the tumor stroma (Figure 5D), but this was less specific at the protein level, with staining detected in other stromal populations. These data illustrate that the stromal compartment acquires the capacity to influence the tumor immune landscape from early stages of development and are dynamic, adapting to the changing requirements of a rapidly growing and evolving tumor (Figure 5E).

To determine whether the identified populations are present in other tumor types, we examined models of murine breast and pancreatic cancers. In orthotopically implanted E0771 breast tumors, the stromal markers distinguishing S1 and S2 from S3 were largely conserved; however, subtle differences in their distribution were detected. While PDPN and PDGFRα colocalized, we also observed some distinction between these markers, as well as a subset of CD34^{+} cells. However, αSMA expression remained more discrete (Figure 5F). In breast tumors, C3 expression was specific to CD34^{high} αSMA^{low} stromal cells at both early and late time points consistent with the melanoma model (Figures 5G, S5D, and S5F; tumor volumes in Figure S5E). Reflecting the temporal dynamics seen in melanoma, αSMA^{high} stromal populations dominated in advanced KPC pancreatic tumors, yet were absent in the normal pancreas. Conversely, pancreatic

Figure 3. T Cells Recruited from Lymph Nodes Are Activated *In Situ*

(A) tSNE plot of individual T cells colored by site (tumor, dark gray; lymph node, light gray) and annotated subpopulations marked by colored lines.

(B) Heatmap showing relative expression (*Z* score) of functional gene groups for cell clusters.

(C) Pseudotime analysis of CD8^{+} T cell gene trajectories colored by site (left), clonal expansion (center), and tumor stage (days, right); arrow indicates time direction.

(D) Expression of activation-associated genes along the inferred pseudotime colored by site; lymph node (green), tumor (blue).

(E) Flow cytometric analysis of T cells isolated from skin and day 5 and 11 tumors, as well as their draining lymph nodes. The number of CD8^{+} cells was quantified, as was proliferation (Ki67) and PD1 expression. Data presented as means ± SEMs, n = 4 independent mice for each condition. *$p < 0.05$, ***$p < 0.001$, ****$p < 0.0001$ (two-way ANOVA with a Sidak post hoc test).

For (A)–(D), n = 10 mice.

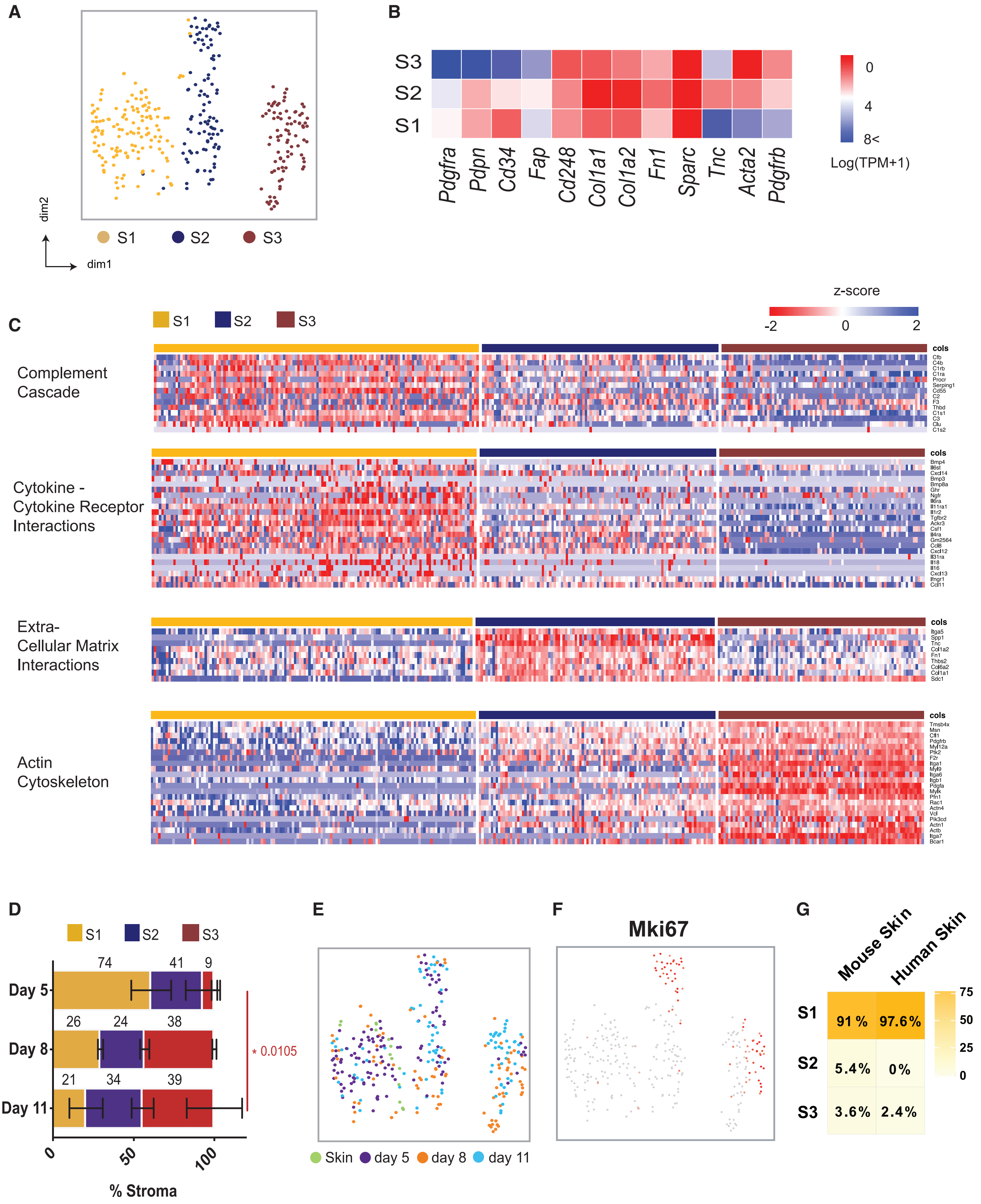

(legend on next page)

stellate cells were predominantly CD34^{high} (Figures S5G–S5J). Publicly available RNA-seq data of KPC-derived CD34^{+} and CD34^{-} stromal populations further showed transcriptional signatures similar to S1 and S2/S3 populations, respectively, as well as CD34^{+}-specific C3 expression (Figures S5K and S5L). These data indicate that major stromal subsets and associated products are preserved across tumor types, albeit with subtle tissue-derived differences.

Crosstalk between the Immune S1 Population and Infiltrating Myeloid Cells

Next, we sought to elucidate the potential functional consequences of specific stromal populations to the ensuing immune response. Focusing on the early S1 immune subset, we examined crosstalk with responsive immune populations recruited to the tumor. To systematically study interactions within the TME, we predicted cell-cell communication networks based on CellPhoneDB, a manually curated repository of ligands, receptors, and their interactions integrated with a statistical framework to infer enriched interactions from single-cell transcriptomic data (Vento-Tormo et al., 2018). This approach highlighted the likely interactions involved in angiogenesis, immune cell recruitment, and immunomodulation between stromal populations in the tumor (Figure 6A; Table S4).

Building on the observations of stromal-derived immunomodulatory factors CSF1, CXCL12, and C3, among others (Figures 5C and 5D), CellPhoneDB identified stromal-immune interactions between C3/CXCL12/CSF1-expressing stromal cells and macrophages positive for C3AR1, CXCR4, and CSFR1, respectively (Figures 6A and 6B). Confocal imaging verified predicted interactions in the tumor stroma, illustrating CSFR1^{+}, CXCR4^{+}, and C3aR^{+} myeloid cells in close contact with CD34^{high} fibroblasts (Figure 6C). The combination of transcriptome profiling and cell-cell communication pipeline enabled us to assign these immune interactions specifically to the S1/S2 subpopulations. Further statistically significant chemokine-receptor interactions occurred between the immune S1 subpopulation, myeloid, Treg, and CD8^{+} T cells (Figure 6A). Intratumoral myeloid populations exhibited the capacity to both attract T cells, via specific cytokine-receptor signals such as CXCL10, CCL22, and CCL5, and suppress their function through the PDL1-PD1 axis (Figure 6A). Our pipeline also predicted interactions between tumor-infiltrating immune populations, including the recruitment of NK cells through cDC1 cell-derived chemokine receptors XCR1 (Böttcher et al., 2018). Moreover, we found that Tregs express high levels of *Nt5e* (CD73) and *Entpd1* (CD39; Figures 3B and 6A), which act together to convert ATP to adenosine, the release of which has been shown to dampen the immune system (Vijayan et al., 2017). Their receptors, *Adora2a* and *Adora2b*, were found to be upregulated on migratory DCs and macrophages, respectively.

Next, we exploited the resource created by our single-cell data and interactions database to explore the functional role of the identified S1-produced candidates. While CXCL12 was expressed by stromal cells, it was also detected within other compartments, including tumor and endothelial cells (Figures 6D and S5A). In contrast, the expression of C3, at the RNA and protein levels, was specific to the S1 population, even in the wider tumor context and in multiple tumor types (Figures 6D, S5A, and S5F). C3 is cleaved to form the anaphylatoxin C3a, which is known to regulate immune populations. Thus, we focused on the consequences of perturbing this C3a-C3aR S1-myeloid interaction within the developing tumor. Neutralization of C3a in established tumors significantly slowed growth compared with immunoglobulin G (IgG) controls (Figure 6E). Furthermore, anti-C3a-treated tumors contained fewer macrophages and more Ly6C^{+} monocytes, specifically at day 6, the point at which C3a-C3aR interactions were predicted to be key mediators of myeloid cell recruitment (Figure 6E). Although the expression of the suppressive molecule PDL1 was not affected by anti-C3a treatment, the density of CD8^{+} T cells (per cubic millimeter) increased at later time points of treated tumors. Similarly, antagonism of the C3a receptor with SB290157 also reduced macrophage infiltration while increasing the number of Ly6C^{+} cells (Figures S6A and S6B). The inversion of F4/80^{+} macrophage and Ly6C^{+} monocyte numbers suggests that the differentiation of infiltrating monocytes to macrophages may be inhibited upon the neutralization of C3a. To corroborate that C3a disruption did not directly affect CD8 T cells, we confirmed the expression of C3aR on both macrophages and Ly6C^{+} myeloid cells, but not T cells at the RNA and protein levels (Figures 6B and S6D). This supports our sequencing data, indicating that the recruitment of suppressive myeloid cells contributes to CD8 T cell suppression. However, other T cell populations were unaffected by C3a neutralization in the TME (Figure S6C). Thus, disrupting signaling between stromal cells and infiltrating myeloid populations has the potential to affect subsequent interactions between the innate and adaptive compartments and promote a better anti-tumor immune response.

Having identified the stromal interactions conserved between murine tumors, we next examined human tumors for the same candidates. We subclustered the stromal compartment of a

Figure 4. Distinct Fibroblast Clusters Identified in Melanoma Tumors

(A) tSNE plot of sequenced CD31^{-} stromal cells from tumors colored by their associated cluster.

(B) Heatmap showing average expression (log(TPM+1)) of typical mesenchymal markers.

(C) Heatmap of Gene Ontology (GO) pathways for differentially expressed genes in each cluster, including cytokine-chemokine receptor interactions, complement cascade, extracellular matrix interactions, and actin cytoskeleton. Columns represent individual cells and rows display *Z* scores.

(D) Sequencing data represented as a bar plot, depicting the ratio of stromal populations at each time point examined. The size of each colored bar is proportional to the percentage of total stromal cells each population represents. Data presented as means ± SEMs, n = 7 mice. *p < 0.05 (two-way ANOVA with Tukey post hoc test).

(E) tSNE plot of sequenced fibroblasts from tumors by tumor time point (right).

(F) tSNE visualization of the proliferation marker Mki67 in the CAFs.

(G) Heatmap depicting logistic regression analysis of normal mouse skin, indicating to which of the 3 stromal clusters these cells are most similar. scRNA-seq of melanoma samples, n = 7 mice. scRNA-seq of healthy murine skin samples, n = 2 mice. scRNA-seq of healthy human skin, n = 1 sample.

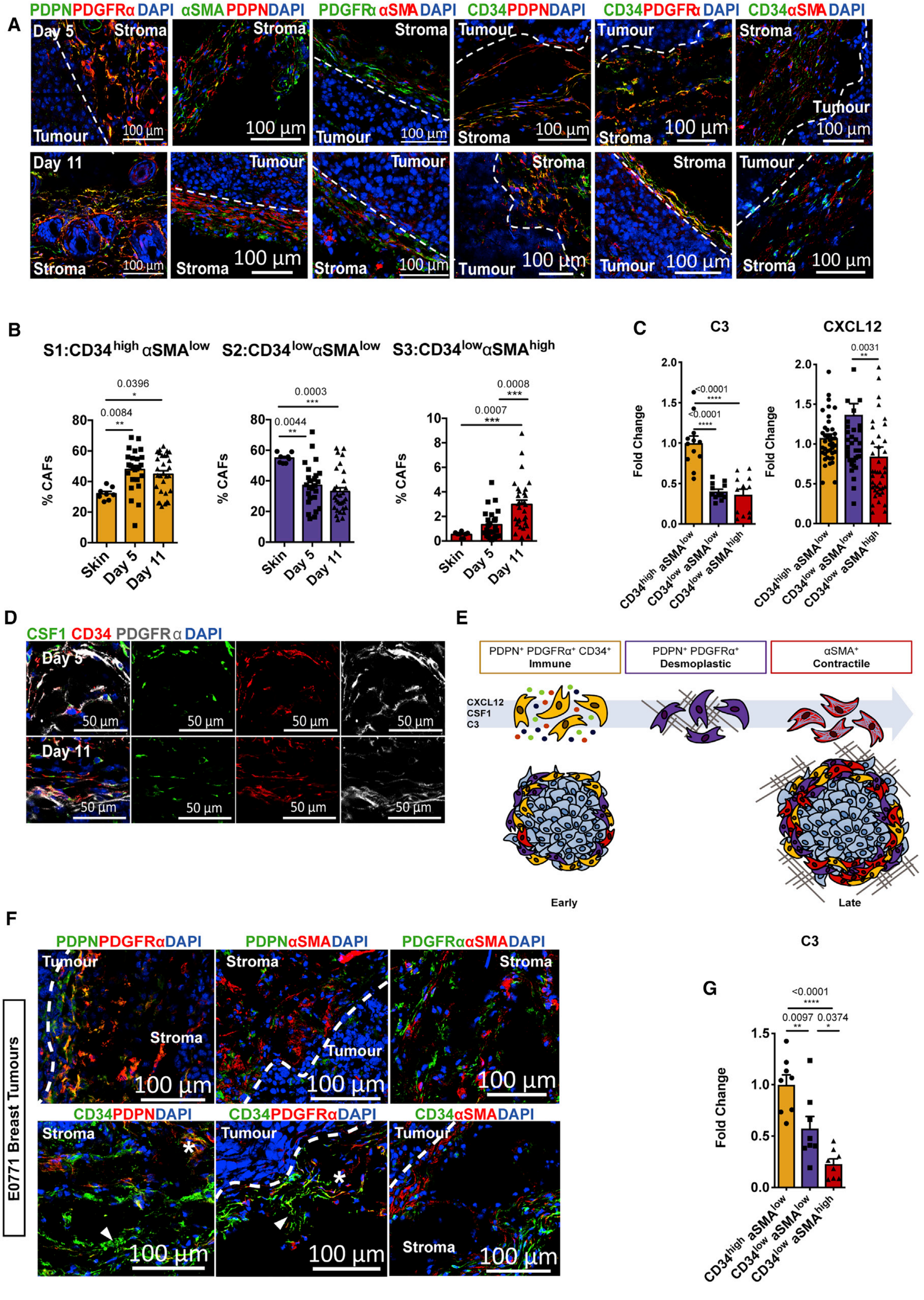

(legend on next page)

human melanoma dataset (Tirosh et al., 2016) and identified distinct populations with markers similar to our S1, S2, and S3 populations (Figures 7A and 7B). Significantly, among the potential immune-stromal interactions identified (Figure 7C), the specific C3a-C3aR interaction between S1 cells and macrophages was retained in human melanoma (Figures 7C and 7D). This interaction was further verified in human head and neck cancer in which the three stromal clusters were also present (Figures 7E–7H). These data indicate a more widespread conservation of CAF-macrophage crosstalk through the C3a-C3aR axis, which translates to multiple tumors and species. Finally, using different The Cancer Genome Atlas (TCGA) datasets, we also demonstrated that in some cancer types, high C3 expression is associated with shorter progression-free survival (Figure S7).

Collectively, these findings provide fundamental insights into the complex interplay among cells within the evolving TME in which multiple immunosuppressive mechanisms coexist (Figure 6F) and highlight the potential of comprehensive datasets to exploit and manipulate CAF-derived immunomodulatory factors found within an increasingly heterogeneous stromal compartment.

DISCUSSION

It is becoming increasingly evident that non-malignant stromal cells provide significant and varied supporting roles as tumors progress. The heterogeneity and dynamic nature of the TME can make identification of the roles of the different immune and stromal components challenging. The emergence of scRNA-seq has enabled deeper insights into tumor biology, revealing the true degree of intratumoral heterogeneity, not detectable by previous methods (Costa et al., 2018; Elyada et al., 2019; Puram et al., 2017; Tirosh et al., 2016). In this study, we used a single-cell transcriptomic approach to characterize the tumor landscape within the changing TME and associated LNs. We identified the gradual development of a suppressive immune microenvironment, specifically in the tumor, as well as discrete stromal subsets with distinct functional signatures. The examination of immune-stromal interactions using the CellPhoneDB database of receptor-ligand interactions (Vento-Tormo et al., 2018) highlighted the complexity of crosstalk between different components of the microenvironment. These interactions were conserved in human tumor tissue.

The immune system, particularly T cells and macrophages, plays key roles in deciding tumor fate and the response to therapy (Angell et al., 2020; Galon and Bruni, 2020; Galon et al., 2006; Sato et al., 2005). Here, we showed site-specific behavior in tumor-associated tissues, identifying distinct gene signatures between populations of the tumor and draining LNs. Pseudotime analysis illustrated that while LNs act as a source of naive T cells, once at the tumor, T cells rapidly transitioned from the naive state through clonal expansion and activation phases (enriched granzyme and IFN expression). As expected, in late stages of tumor growth, T cells upregulated exhaustion markers in late tumors (PD1 and Lag3). The presence of both proliferative and non-cycling exhausted populations indicates an intermediate dysfunctional state, whose exhaustion programs can be reversed before differentiation to a terminally exhausted state (Blank et al., 2019). Similar to T cells, tumor myeloid populations were more activated than their LN counterparts. Coincident with the emergence of T cell dysfunction markers, myeloid cells increased the expression of suppressive factors such as PDL1 and Arg1. This indicates that while LNs act as a T cell reservoir, activation occurs within the tumor itself, followed by the onset of dysfunction markers and myeloid-driven exhaustion in late disease.

While infiltrating immune populations have a profound effect on tumor fate, stromal cells also play a key supporting role in the TME (Kalluri, 2016). However, the development of increasingly diverse populations may underpin conflicting reports regarding anti-tumor versus pro-tumor functions (Feig et al., 2013; Özdemir et al., 2015), making the identification, functional characterization, and tissue-specific features underlying stromal divergence within tumors a priority. Our analysis revealed the existence of three stromal subsets—immune, desmoplastic, and contractile—each possessing unique characteristics indicative of distinct and specialized roles during tumor development. These subsets are in line with recent studies examining the ecosystem of human solid tumors (Bartoschek et al., 2018; Costa et al., 2018; Elyada et al., 2019; Lakins et al., 2018; Lambrechts et al., 2018; Li et al., 2017; Öhlund et al., 2017; Puram et al., 2017). While subtle tissue-specific differences in S1-

Figure 5. Conservation of Fibroblast Subpopulations between Murine Tumor Types

(A) Representative confocal images of PDPN, PDGFRα, and αSMA in combination, or CD34 in combination with either PDPN, PDGFRα, or αSMA (right panel) in day 5 and day 11 tumors. Dashed line indicates the tumor border. Scale bars, 100 μm; images represent at least n = 3 independent mice.

(B) Flow cytometry quantification of the proportion of each stromal population at day 5 and day 11 tumors, displayed as a percentage of the total stromal population. Skin, n = 8 mice; day 5, n = 25 mice; day 11, n = 30 mice.

(C) Flow cytometric quantification of intracellular CXCL12 and C3 expression in each population presented as fold change in mean fluorescence at day 11, normalized to the $CD34^{high}$ αSMA^{low}population. CXCL12, n = 42 tumors; C3, n = 12 tumors.

(D) Representative confocal images of CSF1 expression in $CD34^{+}$ stromal populations in day 5 and day 11 tumors. Scale bars, 50 μm; images represent at least n = 2 independent mice.

(E) Schematic diagram of the 3 stromal subpopulations.

(F) Representative confocal images of stromal population markers in orthotopic E0771 breast tumors. Representative confocal images of PDPN, PDGFRα, and αSMA in combination (top panel) or CD34 in combination with either PDPN, PDGFRα, or αSMA (bottom panel). Dashed line indicates the tumor border. The asterisk indicates colocalization between CD34 and PDPN or PDGFRα; arrowhead indicates CD34 expression that is distinct from PDPN or PDGFRα. Scale bars, 100 μm; images represent at least n = 3 independent mice.

(G) Flow cytometric quantification of intracellular C3 expression in each E0771 breast stromal population presented as fold change in mean fluorescence at day 16 normalized to the $CD34^{high}$ αSMA^{low}. n = 8 independent mice. Each point represents a tumor. Data presented as means ± SEMs. $^{*}p < 0.05$, $^{**}p < 0.01$, $^{***}p < 0.001$, $^{****}p < 0.0001$; one-way ANOVA with a Tukey post hoc test.

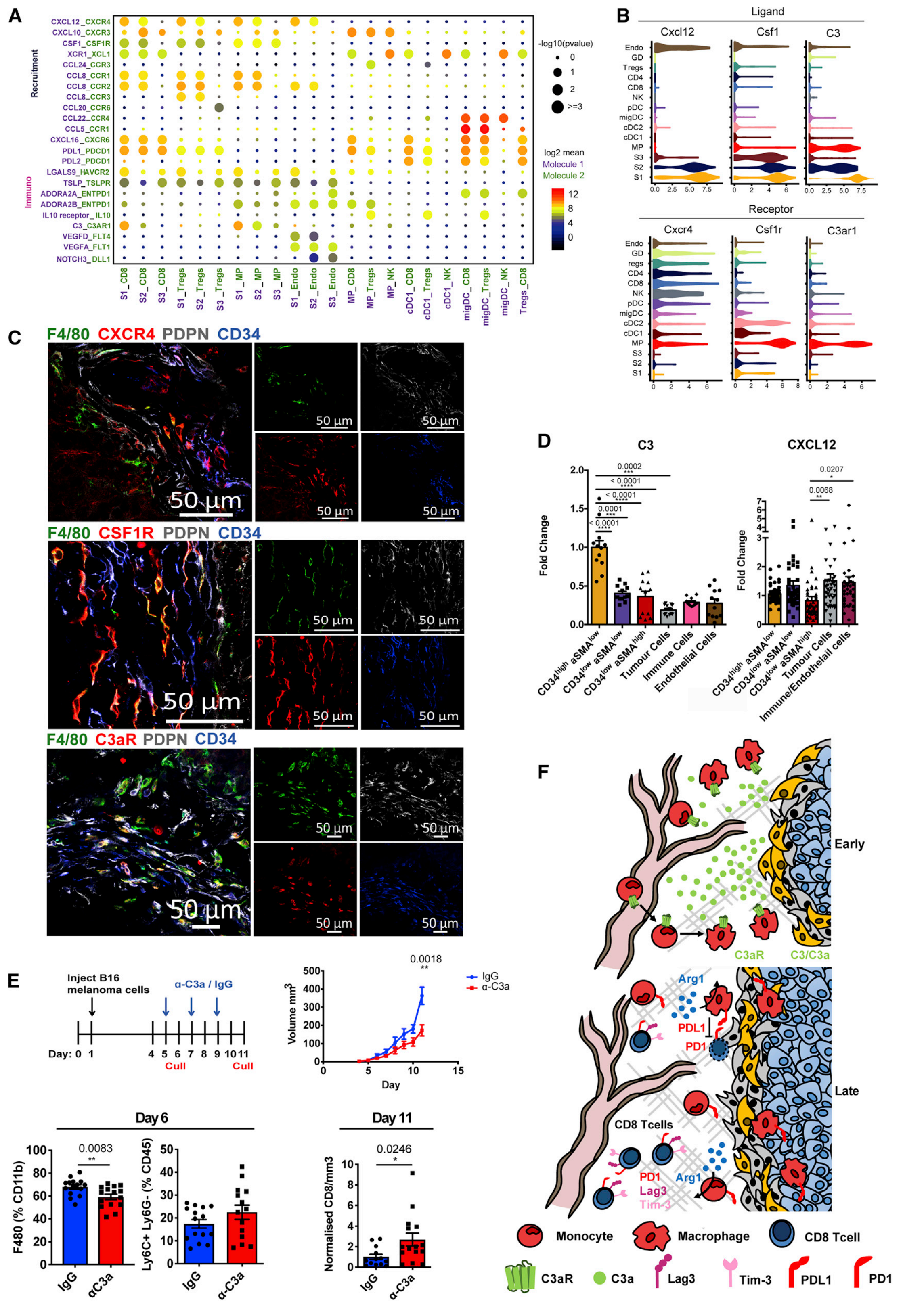

(legend on next page)

associated cytokines and chemokines were seen across cancer types, our data showed that C3 is specifically and consistently upregulated by CD34^{high} populations in multiple mouse models and human datasets. Consistent with other studies, both S3 and LN FRCs shared some typical pericyte markers, such as Rgs5 (Costa et al., 2018; Cremasco et al., 2018; Lambrechts et al., 2018). While this could imply pericyte contamination, S3 also produced significant amounts of matrix components such as *Col1a1*, *Col1a2* (*collagen1*), *Fn1* (*fibronectin1*), and *Sparc*, which are strongly indicative of a fibroblast phenotype. Hence, S3 may embody a mixed population of mesenchymal cells that share similar surface marker expression and functional properties. Alternatively, fibroblasts within S3 may be derived from pericyte populations, as has been observed during fibrosis and tumor development, in which pericytes dissociate from vessels and adopt a myofibroblast phenotype (Chen et al., 2011; Hosaka et al., 2016; Lin et al., 2008; Mederacke et al., 2013),

Our data extend beyond the classification of stromal subsets, providing insight into the kinetics of increasing functional divergence and heterogeneity as the tumor develops. The dominance of S1 and S2 at early time points versus expansion of S3 at later stages is likely the result of events within the adjacent transforming environment, which alter the phenotype, secretory profiles of surrounding cells, and tissue mechanics. Biophysical cues such as matrix rigidity are critical for the maintenance of CAF phenotypes and the induction of αSMA expression (Calvo et al., 2013; Arora et al., 1999; Li et al., 2007). Thus, consistent with inferences from our work and others, the combination of a remodeled and stiffened matrix by desmoplastic S2 fibroblasts and cytokine exposure may induce the expansion of contractile S3 cells in developed tumors (Feig et al., 2013; Raz et al., 2018). For example, in late-stage breast cancer, the expansion of S3-like cells was accompanied by a decrease in matrix-producing fibroblasts (Bartoschek et al., 2018). Such temporal changes may also be supported by the recruitment of mesenchymal cells from the bone marrow (Raz et al., 2018), although this was not the case in B16. Overall, subtle differences in the marker expression, functional properties, and temporal dynamics of fibroblast populations likely reflects the local milieu of soluble factors, mechanical cues, and environmental pressures unique to the tumor type and surrounding tissue at each stage of development.

The sequencing of matched immune and stromal populations enabled us to investigate signaling between different compartments in the TME and highlighted the C3a-C3ar1 axis in S1-myeloid crosstalk. C3 was produced most specifically by CD34^{high} S1 cells, and relevant to the clinic, CD34^{high} CAFs also represented the primary source of C3 in human melanoma and head and neck cancer (Puram et al., 2017; Tirosh et al., 2016). Elevated C3 and C3a have been detected at the primary tumor and in the serum of several solid cancers and are associated with poor prognosis in ovarian cancer (Canales et al., 2014; Chen et al., 2013; Cho et al., 2014; Gast et al., 2009; Habermann et al., 2006). Such results have led to a growing interest in the role of complement activation in cancer, yet little investigation into the source of complement components has been performed. The specific production of C3 by CD34^{high} CAFs indicates that this fibroblast subset may offer a therapeutic target or biomarker application in multiple cancer types. The disruption of C3a-C3aR in tumor-bearing mice slowed tumor growth and reduced infiltrating F4/80^{+} macrophages, which is consistent with studies inhibiting tissue regeneration (Nabizadeh et al., 2016; Zhang et al., 2017). Concurrently, we observed an increase in Ly6C^{+} monocytes, indicating that macrophages are derived from recruited monocytes, which supports a critical role for C3a in monocyte differentiation. The effects observed coincided with increased CD8 T cell numbers per cubic millimeter at later time points. While the inhibition of C3a signaling has been reported to affect T cell phenotypes, we did not detect C3aR on their surface (Kwan et al., 2013; Martin et al., 1997; Quell et al., 2017; Strainic et al., 2013; van der Touw et al., 2013). Thus, alterations in the T cell composition upon C3a neutralization are potentially induced by changes in myeloid populations, implying that the recruitment of macrophages and their products can influence T cell infiltration and behavior. Current trials investigating the efficacy of (human-specific) small-molecule inhibitor compstatin and its derivatives (AMY-101 and APL2) which prevent C3 cleavage (Harris, 2018), may be of interest for tumor therapy in the context of CD34^{high} CAFs. Furthermore, owing to their role in immune regulation, complement therapies have been combined with checkpoint inhibitors (Ajona et al., 2017; Corrales et al., 2012; Markiewski et al., 2008). Thus, it is possible that the stromal-driven inhibitory effects we observed upon the neutralization of C3a may be enhanced if given in combination with immunotherapies.

In summary, we have demonstrated the power of scRNA-seq to define the landscape of the TME and serve as a resource for identifying candidates with therapeutic potential. We identified three

Figure 6. Stromal-Immune Crosstalk Supports the Development of an Immunosuppressive Niche

(A) Overview of selected statistically significant interactions between stromal subsets and other cell types using a cell-cell communication pipeline based on CellPhoneDB. Size indicates p values (permutation test, see STAR Methods), and color indicates the means of the receptor-ligand pairs between 2 clusters.

(B) Violin plots displaying expression log(TPM+1) of ligands *Cxcl12*, *Csf1*, and *C3* and cognate receptors *Cxcr4*, *Csf1r*, and *C3ar1* on respective stromal populations. n = 26 mice.

(C) Confocal images of representative tumor-tissue borders. CXCR4, CSFR1, or C3aR expressing macrophages located proximally to CD34^{+} CAFs (green, F4/80; red, CXCR4, CSF1R, or C3aR; white, PDPN; blue, CD34). Scale bars, 50 μm.

(D) Flow cytometric quantification of CXCL12 and C3 expression across compartments of the tumor microenvironment. Each point represents a tumor. CXCL12 n = 42 tumors, C3 n = 12 tumors. One-way ANOVA with Tukey post hoc test.

(E) *In vivo* blockade of C3a in established tumors. Top left: experimental design and treatment regimen; top right: tumor volume (in cubic millimeters) of mice treated with IgG control (blue) or anti-C3a (red); bottom left: myeloid infiltration in day 6 tumors, after 24 h of treatment with IgG or anti-C3a. The number of F4/80 and Ly6C^{+} Ly6G^{-} cells are shown as a percentage of Cd11b and CD45 cells, respectively; bottom right: the number of tumor-infiltrating CD8^{+} T cells at day 11, displayed as raw counts normalized to tumor volume (in cubic millimeters). Data presented as means ± SEMs. n = minimum 13 mice. **p < 0.01, ***p < 0.001, ****p < 0.0001; t test.

(F) Schematic diagram of the dynamic crosstalk identified within the tumor microenvironment.

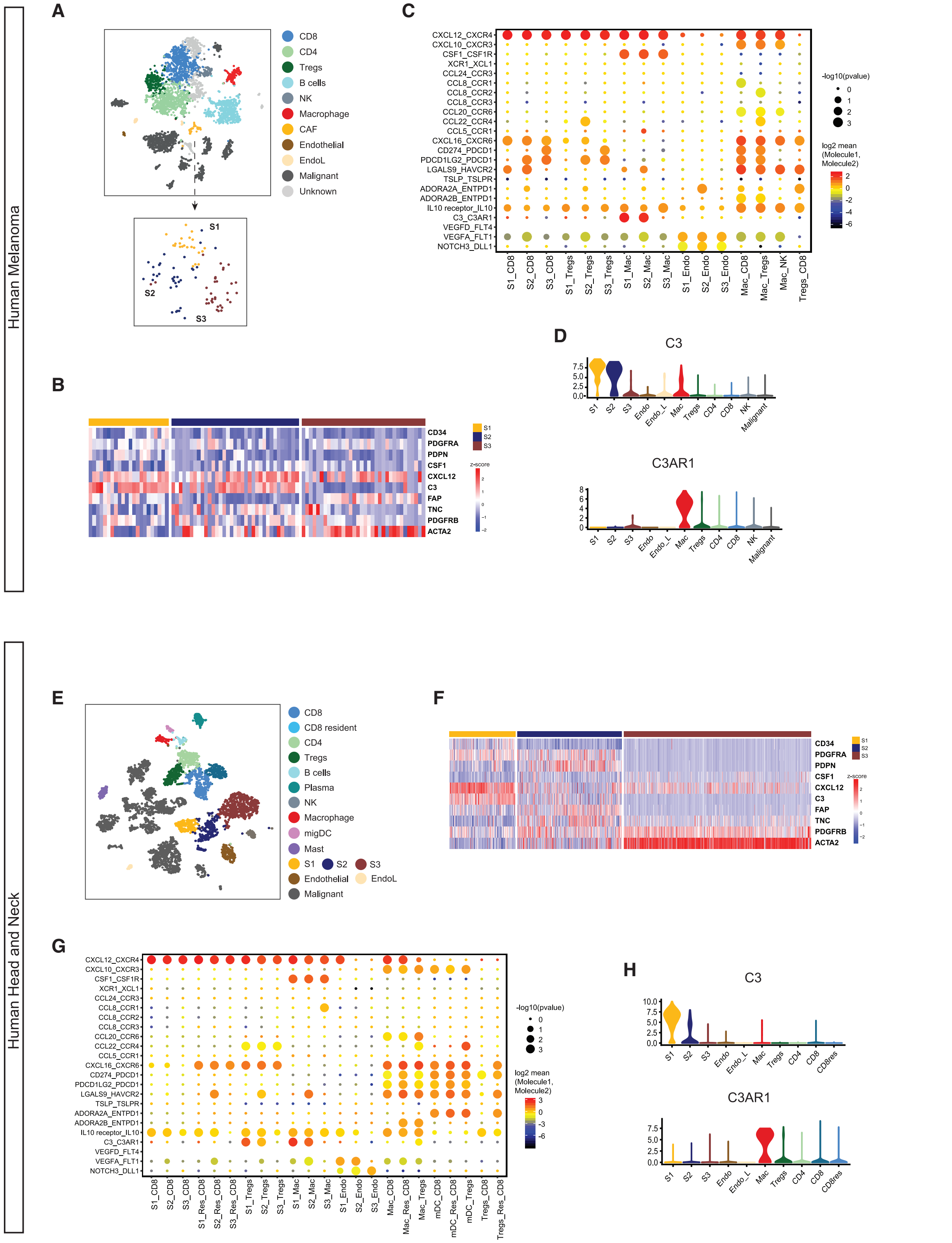

(legend on next page)

stromal clusters with distinct functional and temporal features, highlighting the dynamic and adaptive nature of both immune and stromal populations to reveal potential crosstalk between these two compartments. By supporting the recruitment and induction of an immunosuppressive macrophage phenotype, the immune stromal subset may provide an alternative, indirect mechanism to dampen T cell-mediated anti-tumor immunity.

STAR★METHODS

Detailed methods are provided in the online version of this paper and include the following:

- KEY RESOURCES TABLE
- LEAD CONTACT AND MATERIALS AVAILABILITY
- EXPERIMENTAL MODEL AND SUBJECT DETAILS
  - Cell lines
  - Mice
  - Orthotopic syngeneic tumor models
  - Neutralising C3a/C3aR *in vivo*
  - EdU Incorporation
- METHOD DETAILS
  - Tissue Processing
  - Isolation of Single Cells
  - Preparation of cDNA and sequencing
  - Flow Cytometry
  - Immunofluorescence
- QUANTIFICATION AND STATISTICAL ANALYSIS
  - Single-cell RNA sequencing analysis
  - T cell receptor (TCR) analysis
  - Cell cycle analysis
  - Putative interactions between cell types
  - Mouse skin fibroblasts from healthy mice
  - Human skin fibroblasts
  - Comparison of human and mouse skin fibroblasts with stromal clusters
  - Public data analysis
- DATA AND CODE AVAILABILITY

SUPPLEMENTAL INFORMATION

Supplemental Information can be found online at https://doi.org/10.1016/j.celrep.2020.107628.

ACKNOWLEDGMENTS

We thank all of the members of the Teichmann and Shields lab for helpful discussion and the staff at the Medical Research Council ARES facility and Cambridge Institute for Medical Research Flow Cytometry Core. This project was supported by the Cancer Research UK Cancer Immunology Fund (ref. 20193), European Research Council grants (ThSWITCH, grant no. 260507; ThDEFINE, Project ID 646794), a European Union Future and Emerging Technologies (FET)-Open grant (MRG-GRAMMAR no. 664918), Wellcome Sanger core funding (no. WT206194), and Medical Research Council core funding (MC_UU_12022/5).

AUTHOR CONTRIBUTIONS

S.A.T., J.D.S., and B.M. conceived the study. S.D., A.R., B.M., J.D.S., and J.P. performed the mouse experiments, the sample/library preparation, and the histology staining. M.E. and S.D. analyzed the data and interpreted the results, with contributions from J.H., G.K., S.A.T., J.D.S., and R.V.-T. T.H. and X.C. performed the sample/library preparation for the healthy mouse skin samples. S.D., M.E., J.D.S., and S.A.T. wrote the manuscript. S.A.T. and J.D.S. co-directed the study. All of the authors read and accepted the manuscript.

DECLARATION OF INTERESTS

The authors declare no competing interests.

Received: August 29, 2019
Revised: January 22, 2020
Accepted: April 18, 2020
Published: May 19, 2020

REFERENCES

Ajona, D., Ortiz-Espinosa, S., Moreno, H., Lozano, T., Pajares, M.J., Agorreta, J., Bértolo, C., Lasarte, J.J., Vicent, S., Hoehlig, K., et al. (2017). A Combined PD-1/C5a Blockade Synergistically Protects against Lung Cancer Growth and Metastasis. Cancer Discov. *7*, 694–703.

Angell, H.K., Bruni, D., Barrett, J.C., Herbst, R., and Galon, J. (2020). The Immunoscore: Colon Cancer and Beyond. Clin. Cancer Res. *26*, 332–339.

Arora, P.D., Narani, N., and McCulloch, C.A. (1999). The compliance of collagen gels regulates transforming growth factor-beta induction of alpha-smooth muscle actin in fibroblasts. Am. J. Pathol. *154*, 871–882.

Augsten, M. (2014). Cancer-associated fibroblasts as another polarized cell type of the tumor microenvironment. Front. Oncol. *4*, 62.

Bartoschek, M., Oskolkov, N., Bocci, M., Lövrot, J., Larsson, C., Sommarin, M., Madsen, C.D., Lindgren, D., Pekar, G., Karlsson, G., et al. (2018). Spatially and functionally distinct subclasses of breast cancer-associated fibroblasts revealed by single cell RNA sequencing. Nat. Commun. *9*, 5150.

Figure 7. Similar Stromal Populations and C3-C3aR Interactions Are Conserved in Human Melanoma and Head and Neck Cancer
Publically available single-cell sequencing data from human melanoma and head and neck cancer were downloaded and analyzed.
(A) tSNE plots of sequenced populations for melanoma.
(B) Heatmap depicting stromal subsets 1–3 defined by similar markers and functional features to murine melanoma dataset. Heatmap displays the expression (*Z* scores, blue to red) of key markers and cytokines across stromal clusters identified in human melanoma.
(C) Overview of statistically significant interactions between stromal subsets and other cell types using the CellPhoneDB pipeline. Size indicates p values and color indicates the means of the receptor-ligand pairs between 2 clusters.
(D) Violin plots displaying conserved expression log(TPM+1) of *C3* and cognate receptor *C3ar1* on respective stromal populations in human melanoma. n = 19 patient samples.
(E) tSNE plots of sequenced populations for human head and neck cancer.
(F) Heatmap depicting stromal subsets 1–3 defined by similar markers and functional features to murine melanoma dataset. Heatmap displays expression (*Z* scores, blue to red) of key markers and cytokines across stromal clusters identified in human head and neck cancer.
(G) Statistically significant interactions between stromal subsets and other cell types using the CellPhoneDB pipeline. Size indicates p values and color indicates the means of the receptor-ligand pairs between 2 clusters.
(H) Violin plots displaying conserved expression log(TPM+1) of *C3* and cognate receptor *C3ar1* on stromal populations in human head and neck cancer. n = 18 patient samples.

Blank, C.U., Haining, W.N., Held, W., Hogan, P.G., Kallies, A., Lugli, E., Lynn, R.C., Philip, M., Rao, A., Restifo, N.P., et al. (2019). Defining 'T cell exhaustion'. Nat. Rev. Immunol. *19*, 665–674.

Bonnans, C., Chou, J., and Werb, Z. (2014). Remodelling the extracellular matrix in development and disease. Nat. Rev. Mol. Cell Biol. *15*, 786–801.

Böttcher, J.P., Bonavita, E., Chakravarty, P., Blees, H., Cabeza-Cabrerizo, M., Sammicheli, S., Rogers, N.C., Sahai, E., Zelenay, S., and Reis e Sousa, C. (2018). NK Cells Stimulate Recruitment of cDC1 into the Tumor Microenvironment Promoting Cancer Immune Control. Cell *172*, 1022–1037.e14.

Brahmer, J.R., Tykodi, S.S., Chow, L.Q.M., Hwu, W.-J., Topalian, S.L., Hwu, P., Drake, C.G., Camacho, L.H., Kauh, J., Odunsi, K., et al. (2012). Safety and activity of anti-PD-L1 antibody in patients with advanced cancer. N. Engl. J. Med. *366*, 2455–2465.

Calon, A., Espinet, E., Palomo-Ponce, S., Tauriello, D.V.F., Iglesias, M., Céspedes, M.V., Sevillano, M., Nadal, C., Jung, P., Zhang, X.H.-F., et al. (2012). Dependency of colorectal cancer on a TGF-β-driven program in stromal cells for metastasis initiation. Cancer Cell *22*, 571–584.

Calvo, F., Ege, N., Grande-Garcia, A., Hooper, S., Jenkins, R.P., Chaudhry, S.I., Harrington, K., Williamson, P., Moeendarbary, E., Charras, G., and Sahai, E. (2013). Mechanotransduction and YAP-dependent matrix remodelling is required for the generation and maintenance of cancer-associated fibroblasts. Nat. Cell Biol. *15*, 637–646.

Canales, N.A.G., Marina, V.M., Castro, J.S., Jiménez, A.A., Mendoza-Hernández, G., McCarron, E.L., Roman, M.B., and Castro-Romero, J.I. (2014). A1BG and C3 are overexpressed in patients with cervical intraepithelial neoplasia III. Oncol. Lett. *8*, 939–947.

Cerami, E., Gao, J., Dogrusoz, U., Gross, B.E., Sumer, S.O., Aksoy, B.A., Jacobsen, A., Byrne, C.J., Heuer, M.L., Larsson, E., et al. (2012). The cBio cancer genomics portal: an open platform for exploring multidimensional cancer genomics data. Cancer Discov. *2*, 401–404.

Chen, Y.-T., Chang, F.-C., Wu, C.-F., Chou, Y.-H., Hsu, H.-L., Chiang, W.-C., Shen, J., Chen, Y.-M., Wu, K.-D., Tsai, T.-J., et al. (2011). Platelet-derived growth factor receptor signaling activates pericyte-myofibroblast transition in obstructive and post-ischemic kidney fibrosis. Kidney Int. *80*, 1170–1181.

Chen, J., Wu, W., Chen, L., Zhou, H., Yang, R., Hu, L., and Zhao, Y. (2013). Profiling the potential tumor markers of pancreatic ductal adenocarcinoma using 2D-DIGE and MALDI-TOF-MS: up-regulation of Complement C3 and alpha-2-HS-glycoprotein. Pancreatology *13*, 290–297.

Cho, M.S., Vasquez, H.G., Rupaimoole, R., Pradeep, S., Wu, S., Zand, B., Han, H.-D., Rodriguez-Aguayo, C., Bottsford-Miller, J., Huang, J., et al. (2014). Autocrine effects of tumor-derived complement. Cell Rep. *6*, 1085–1095.

Colaprico, A., Silva, T.C., Olsen, C., Garofano, L., Cava, C., Garolini, D., Sabedot, T.S., Malta, T.M., Pagnotta, S.M., Castiglioni, I., et al. (2016). TCGAbiolinks: an R/Bioconductor package for integrative analysis of TCGA data. Nucleic Acids Res. *44*, e71.

Corrales, L., Ajona, D., Rafail, S., Lasarte, J.J., Riezu-Boj, J.I., Lambris, J.D., Rouzaut, A., Pajares, M.J., Montuenga, L.M., and Pio, R. (2012). Anaphylatoxin C5a creates a favorable microenvironment for lung cancer progression. J. Immunol. *189*, 4674–4683.

Cortez, E., Roswall, P., and Pietras, K. (2014). Functional subsets of mesenchymal cell types in the tumor microenvironment. Semin. Cancer Biol. *25*, 3–9.

Costa, A., Kieffer, Y., Scholer-Dahirel, A., Pelon, F., Bourachot, B., Cardon, M., Sirven, P., Magagna, I., Fuhrmann, L., Bernard, C., et al. (2018). Fibroblast Heterogeneity and Immunosuppressive Environment in Human Breast Cancer. Cancer Cell *33*, 463–479.e10.

Cremasco, V., Astarita, J.L., Grauel, A.L., Keerthivasan, S., MacIsaac, K., Woodruff, M.C., Wu, M., Spel, L., Santoro, S., Amoozgar, Z., et al. (2018). FAP Delineates Heterogeneous and Functionally Divergent Stromal Cells in Immune-Excluded Breast Tumors. Cancer Immunol. Res. *6*, 1472–1485.

Direkze, N.C., Hodivala-Dilke, K., Jeffery, R., Hunt, T., Poulsom, R., Oukrif, D., Alison, M.R., and Wright, N.A. (2004). Bone marrow contribution to tumor-associated myofibroblasts and fibroblasts. Cancer Res. *64*, 8492–8495.

Dumont, N., Liu, B., Defilippis, R.A., Chang, H., Rabban, J.T., Karnezis, A.N., Tjoe, J.A., Marx, J., Parvin, B., and Tlsty, T.D. (2013). Breast fibroblasts modulate early dissemination, tumorigenesis, and metastasis through alteration of extracellular matrix characteristics. Neoplasia *15*, 249–262.

Elyada, E., Bolisetty, M., Laise, P., Flynn, W.F., Courtois, E.T., Burkhart, R.A., Teinor, J.A., Belleau, P., Biffi, G., Lucito, M.S., et al. (2019). Cross-Species Single-Cell Analysis of Pancreatic Ductal Adenocarcinoma Reveals Antigen-Presenting Cancer-Associated Fibroblasts. Cancer Discov. *9*, 1102–1123.

Feig, C., Jones, J.O., Kraman, M., Wells, R.J.B., Deonarine, A., Chan, D.S., Connell, C.M., Roberts, E.W., Zhao, Q., Caballero, O.L., et al. (2013). Targeting CXCL12 from FAP-expressing carcinoma-associated fibroblasts synergizes with anti-PD-L1 immunotherapy in pancreatic cancer. Proc. Natl. Acad. Sci. USA *110*, 20212–20217.

Gaggioli, C., Hooper, S., Hidalgo-Carcedo, C., Grosse, R., Marshall, J.F., Harrington, K., and Sahai, E. (2007). Fibroblast-led collective invasion of carcinoma cells with differing roles for RhoGTPases in leading and following cells. Nat. Cell Biol. *9*, 1392–1400.

Galon, J., and Bruni, D. (2020). Tumor Immunology and Tumor Evolution: Intertwined Histories. Immunity *52*, 55–81.

Galon, J., Costes, A., Sanchez-Cabo, F., Kirilovsky, A., Mlecnik, B., Lagorce-Pagès, C., Tosolini, M., Camus, M., Berger, A., Wind, P., et al. (2006). Type, density, and location of immune cells within human colorectal tumors predict clinical outcome. Science *313*, 1960–1964.

Gao, J., Aksoy, B.A., Dogrusoz, U., Dresdner, G., Gross, B., Sumer, S.O., Sun, Y., Jacobsen, A., Sinha, R., Larsson, E., et al. (2013). Integrative analysis of complex cancer genomics and clinical profiles using the cBioPortal. Sci. Signal. *6*, pl1.

Gast, M.-C.W., Van Gils, C.H., Wessels, L.F.A., Harris, N., Bonfrer, J.M.G., Rutgers, E.J.T., Schellens, J.H.M., and Beijnen, J.H. (2009). Serum protein profiling for diagnosis of breast cancer using SELDI-TOF MS. Oncol. Rep. *22*, 205–213.

Guo, X., Oshima, H., Kitmura, T., Taketo, M.M., and Oshima, M. (2008). Stromal fibroblasts activated by tumor cells promote angiogenesis in mouse gastric cancer. J. Biol. Chem. *283*, 19864–19871.

Habermann, J.K., Roblick, U.J., Luke, B.T., Prieto, D.A., Finlay, W.J.J., Podust, V.N., Roman, J.M., Oevermann, E., Schiedeck, T., Homann, N., et al. (2006). Increased serum levels of complement C3a anaphylatoxin indicate the presence of colorectal tumors. Gastroenterology *131*, 1020–1029.

Hagai, T., Chen, X., Miragaia, R.J., Rostom, R., Gomes, T., Kunowska, N., Henriksson, J., Park, J.-E., Proserpio, V., Donati, G., et al. (2018). Gene expression variability across cells and species shapes innate immunity. Nature *563*, 197–202.

Hamid, O., Robert, C., Daud, A., Hodi, F.S., Hwu, W.-J., Kefford, R., Wolchok, J.D., Hersey, P., Joseph, R.W., Weber, J.S., et al. (2013). Safety and tumor responses with lambrolizumab (anti-PD-1) in melanoma. N. Engl. J. Med. *369*, 134–144.

Harper, J., and Sainson, R.C.A. (2014). Regulation of the anti-tumour immune response by cancer-associated fibroblasts. Semin. Cancer Biol. *25*, 69–77.

Harris, C.L. (2018). Expanding horizons in complement drug discovery: challenges and emerging strategies. Semin. Immunopathol. *40*, 125–140.

Hodi, F.S., O'Day, S.J., McDermott, D.F., Weber, R.W., Sosman, J.A., Haanen, J.B., Gonzalez, R., Robert, C., Schadendorf, D., Hassel, J.C., et al. (2010). Improved survival with ipilimumab in patients with metastatic melanoma. N. Engl. J. Med. *363*, 711–723.

Hosaka, K., Yang, Y., Seki, T., Fischer, C., Dubey, O., Fredlund, E., Hartman, J., Religa, P., Morikawa, H., Ishii, Y., et al. (2016). Pericyte-fibroblast transition promotes tumor growth and metastasis. Proc. Natl. Acad. Sci. USA *113*, E5618–E5627.

Jia, C.-C., Wang, T.-T., Liu, W., Fu, B.-S., Hua, X., Wang, G.-Y., Li, T.-J., Li, X., Wu, X.-Y., Tai, Y., et al. (2013). Cancer-associated fibroblasts from hepatocellular carcinoma promote malignant cell proliferation by HGF secretion. PLoS One *8*, e63243.

CellPress
OPEN ACCESS

Junttila, M.R., and de Sauvage, F.J. (2013). Influence of tumour micro-environment heterogeneity on therapeutic response. Nature *501*, 346–354.

Kalluri, R. (2016). The biology and function of fibroblasts in cancer. Nat. Rev. Cancer *16*, 582–598.

Kwan, W.-H., van der Touw, W., Paz-Artal, E., Li, M.O., and Heeger, P.S. (2013). Signaling through C5a receptor and C3a receptor diminishes function of murine natural regulatory T cells. J. Exp. Med. *210*, 257–268.

La Manno, G., Gyllborg, D., Codeluppi, S., Nishimura, K., Salto, C., Zeisel, A., Borm, L.E., Stott, S.R.W., Toledo, E.M., Villaescusa, J.C., et al. (2016). Molecular Diversity of Midbrain Development in Mouse, Human, and Stem Cells. Cell *167*, 566–580.e19.

Lakins, M.A., Ghorani, E., Munir, H., Martins, C.P., and Shields, J.D. (2018). Cancer-associated fibroblasts induce antigen-specific deletion of CD8 $^{+}$ T Cells to protect tumour cells. Nat. Commun. *9*, 948.

Lambrechts, D., Wauters, E., Boeckx, B., Aibar, S., Nittner, D., Burton, O., Bassez, A., Decaluwé, H., Pircher, A., Van den Eynde, K., et al. (2018). Phenotype molding of stromal cells in the lung tumor microenvironment. Nat. Med. *24*, 1277–1289.

Li, Z., Dranoff, J.A., Chan, E.P., Uemura, M., Sévigny, J., and Wells, R.G. (2007). Transforming growth factor-beta and substrate stiffness regulate portal fibroblast activation in culture. Hepatology *46*, 1246–1256.

Li, H., Courtois, E.T., Sengupta, D., Tan, Y., Chen, K.H., Goh, J.J.L., Kong, S.L., Chua, C., Hon, L.K., Tan, W.S., et al. (2017). Reference component analysis of single-cell transcriptomes elucidates cellular heterogeneity in human colorectal tumors. Nat. Genet. *49*, 708–718.

Li, H., van der Leun, A.M., Yofe, I., Lubling, Y., Gelbard-Solodkin, D., van Akkooi, A.C.J., van den Braber, M., Rozeman, E.A., Haanen, J.B.A.G., Blank, C.U., et al. (2019). Dysfunctional CD8 T Cells Form a Proliferative, Dynamically Regulated Compartment within Human Melanoma. Cell *176*, 775–789.e18.

Lin, S.-L., Kisseleva, T., Brenner, D.A., and Duffield, J.S. (2008). Pericytes and perivascular fibroblasts are the primary source of collagen-producing cells in obstructive fibrosis of the kidney. Am. J. Pathol. *173*, 1617–1627.

Love, M.I., Huber, W., and Anders, S. (2014). Moderated estimation of fold change and dispersion for RNA-seq data with DESeq2. Genome Biol. *15*, 550.

Markiewski, M.M., DeAngelis, R.A., Benencia, F., Ricklin-Lichtsteiner, S.K., Koutoulaki, A., Gerard, C., Coukos, G., and Lambris, J.D. (2008). Modulation of the antitumor immune response by complement. Nat. Immunol. *9*, 1225–1235.

Martin, U., Bock, D., Arseniev, L., Tornetta, M.A., Ames, R.S., Bautsch, W., Köhl, J., Ganser, A., and Klos, A. (1997). The human C3a receptor is expressed on neutrophils and monocytes, but not on B or T lymphocytes. J. Exp. Med. *186*, 199–207.

Mederacke, I., Hsu, C.C., Troeger, J.S., Huebener, P., Mu, X., Dapito, D.H., Pradere, J.-P., and Schwabe, R.F. (2013). Fate tracing reveals hepatic stellate cells as dominant contributors to liver fibrosis independent of its aetiology. Nat. Commun. *4*, 2823.

Nabizadeh, J.A., Manthey, H.D., Steyn, F.J., Chen, W., Widiapradja, A., Md Akhir, F.N., Boyle, G.M., Taylor, S.M., Woodruff, T.M., and Rolfe, B.E. (2016). The Complement C3a Receptor Contributes to Melanoma Tumorigenesis by Inhibiting Neutrophil and CD4+ T Cell Responses. J. Immunol. *196*, 4783–4792.

Öhlund, D., Handly-Santana, A., Biffi, G., Elyada, E., Almeida, A.S., Ponz-Sarvise, M., Corbo, V., Oni, T.E., Hearn, S.A., Lee, E.J., et al. (2017). Distinct populations of inflammatory fibroblasts and myofibroblasts in pancreatic cancer. J. Exp. Med. *214*, 579–596.

Orimo, A., Gupta, P.B., Sgroi, D.C., Arenzana-Seisdedos, F., Delaunay, T., Naeem, R., Carey, V.J., Richardson, A.L., and Weinberg, R.A. (2005). Stromal fibroblasts present in invasive human breast carcinomas promote tumor growth and angiogenesis through elevated SDF-1/CXCL12 secretion. Cell *121*, 335–348.

Özdemir, B.C., Pentcheva-Hoang, T., Carstens, J.L., Zheng, X., Wu, C.-C., Simpson, T.R., Laklai, H., Sugimoto, H., Kahlert, C., Novitskiy, S.V., et al. (2015). Depletion of Carcinoma-Associated Fibroblasts and Fibrosis Induces Immunosuppression and Accelerates Pancreas Cancer with Reduced Survival. Cancer Cell *28*, 831–833.

Patro, R., Duggal, G., Love, M.I., Irizarry, R.A., and Kingsford, C. (2017). Salmon provides fast and bias-aware quantification of transcript expression. Nat. Methods *14*, 417–419.

Picelli, S., Faridani, O.R., Björklund, A.K., Winberg, G., Sagasser, S., and Sandberg, R. (2014). Full-length RNA-seq from single cells using Smart-seq2. Nat. Protoc. *9*, 171–181.

Puram, S.V., Tirosh, I., Parikh, A.S., Patel, A.P., Yizhak, K., Gillespie, S., Rodman, C., Luo, C.L., Mroz, E.A., Emerick, K.S., et al. (2017). Single-Cell Transcriptomic Analysis of Primary and Metastatic Tumor Ecosystems in Head and Neck Cancer. Cell *171*, 1611–1624.e24.

Qiu, X., Mao, Q., Tang, Y., Wang, L., Chawla, R., Pliner, H.A., and Trapnell, C. (2017). Reversed graph embedding resolves complex single-cell trajectories. Nat. Methods *14*, 979–982.

Quante, M., Tu, S.P., Tomita, H., Gonda, T., Wang, S.S.W., Takashi, S., Baik, G.H., Shibata, W., Diprete, B., Betz, K.S., et al. (2011). Bone marrow-derived myofibroblasts contribute to the mesenchymal stem cell niche and promote tumor growth. Cancer Cell *19*, 257–272.

Quell, K.M., Karsten, C.M., Kordowski, A., Almeida, L.N., Briukhovetska, D., Wiese, A.V., Sun, J., Ender, F., Antoniou, K., Schröder, T., et al. (2017). Monitoring C3aR Expression Using a Floxed tdTomato-C3aR Reporter Knock-in Mouse. J. Immunol. *199*, 688–706.

Raudvere, U., Kolberg, L., Kuzmin, I., Arak, T., Adler, P., Peterson, H., and Vilo, J. (2019). g:Profiler: a web server for functional enrichment analysis and conversions of gene lists (2019 update). Nucleic Acids Res. *47* (*W1*), W191–W198.

Raz, Y., Cohen, N., Shani, O., Bell, R.E., Novitskiy, S.V., Abramovitz, L., Levy, C., Milyavsky, M., Leider-Trejo, L., Moses, H.L., et al. (2018). Bone marrow-derived fibroblasts are a functionally distinct stromal cell population in breast cancer. J. Exp. Med. *215*, 3075–3093.

Roswall, P., and Pietras, K. (2012). Of mice and men: a comparative study of cancer-associated fibroblasts in mammary carcinoma. Ups. J. Med. Sci. *117*, 196–201.

Satija, R., Farrell, J.A., Gennert, D., Schier, A.F., and Regev, A. (2015). Spatial reconstruction of single-cell gene expression data. Nat. Biotechnol. *33*, 495–502.

Sato, E., Olson, S.H., Ahn, J., Bundy, B., Nishikawa, H., Qian, F., Jungbluth, A.A., Frosina, D., Gnjatic, S., Ambrosone, C., et al. (2005). Intraepithelial CD8+ tumor-infiltrating lymphocytes and a high CD8+/regulatory T cell ratio are associated with favorable prognosis in ovarian cancer. Proc. Natl. Acad. Sci. USA *102*, 18538–18543.

Scialdone, A., Natarajan, K.N., Saraiva, L.R., Proserpio, V., Teichmann, S.A., Stegle, O., Marioni, J.C., and Buettner, F. (2015). Computational assignment of cell-cycle stage from single-cell transcriptome data. Methods *85*, 54–61.

Simoni, Y., Becht, E., Fehlings, M., Loh, C.Y., Koo, S.-L., Teng, K.W.W., Yeong, J.P.S., Nahar, R., Zhang, T., Kared, H., et al. (2018). Bystander CD8^{+} T cells are abundant and phenotypically distinct in human tumour infiltrates. Nature *557*, 575–579.

Strainic, M.G., Shevach, E.M., An, F., Lin, F., and Medof, M.E. (2013). Absence of signaling into CD4^{+} cells via C3aR and C5aR enables autoinductive TGF-β1 signaling and induction of Foxp3^{+} regulatory T cells. Nat. Immunol. *14*, 162–171.

Stubbington, M.J.T., Lönnberg, T., Proserpio, V., Clare, S., Speak, A.O., Dougan, G., and Teichmann, S.A. (2016). T cell fate and clonality inference from single-cell transcriptomes. Nat. Methods *13*, 329–332.

Therneau, T.M., and Grambsch, P.M. (2000). Modeling Survival Data: Extending the Cox Model (Springer).

Tirosh, I., Izar, B., Prakadan, S.M., Wadsworth, M.H., 2nd, Treacy, D., Trombetta, J.J., Rotem, A., Rodman, C., Lian, C., Murphy, G., et al. (2016). Dissecting the multicellular ecosystem of metastatic melanoma by single-cell RNA-seq. Science *352*, 189–196.

Topalian, S.L., Hodi, F.S., Brahmer, J.R., Gettinger, S.N., Smith, D.C., McDermott, D.F., Powderly, J.D., Carvajal, R.D., Sosman, J.A., Atkins, M.B., et al.

(2012). Safety, activity, and immune correlates of anti-PD-1 antibody in cancer. N. Engl. J. Med. *366*, 2443–2454.

Turley, S.J., Cremasco, V., and Astarita, J.L. (2015). Immunological hallmarks of stromal cells in the tumour microenvironment. Nat. Rev. Immunol. *15*, 669–682.

van der Touw, W., Cravedi, P., Kwan, W.-H., Paz-Artal, E., Merad, M., and Heeger, P.S. (2013). Cutting edge: receptors for C3a and C5a modulate stability of alloantigen-reactive induced regulatory T cells. J. Immunol. *190*, 5921–5925.

Vento-Tormo, R., Efremova, M., Botting, R.A., Turco, M.Y., Vento-Tormo, M., Meyer, K.B., Park, J.-E., Stephenson, E., Polański, K., Goncalves, A., et al. (2018). Single-cell reconstruction of the early maternal-fetal interface in humans. Nature *563*, 347–353.

Vijayan, D., Young, A., Teng, M.W.L., and Smyth, M.J. (2017). Targeting immunosuppressive adenosine in cancer. Nat. Rev. Cancer *17*, 709–724.

Zhang, C., Wang, C., Li, Y., Miwa, T., Liu, C., Cui, W., Song, W.-C., and Du, J. (2017). Complement C3a signaling facilitates skeletal muscle regeneration by regulating monocyte function and trafficking. Nat. Commun. *8*, 2078.

Zheng, G.X.Y., Terry, J.M., Belgrader, P., Ryvkin, P., Bent, Z.W., Wilson, R., Ziraldo, S.B., Wheeler, T.D., McDermott, G.P., Zhu, J., et al. (2017). Massively parallel digital transcriptional profiling of single cells. Nat. Commun. *8*, 14049.

STAR★METHODS

KEY RESOURCES TABLE

REAGENT or RESOURCE	SOURCE	IDENTIFIER
Antibodies		
Rat CD45 APC-Cy7	Biolegend	Cat# 103116, RRID:AB_312981
Rat CD45 FITC	Biolegend	Cat# 103108, RRID:AB_312973
Rat CD45 BV785	Biolegend	Cat# 103149, RRID:AB_2564590
Rat CD31 PE-Cy7	eBioscience	Cat# 102417, RRID:AB_830756
Rat CD31 biotin	eBioscience	Cat# 102503, RRID:AB_312910
Syrian Hamster PDPN APC	Biolegend	Cat# 127410, RRID:AB_10613649
Armenian Hamster CD3e 488	Biolegend	Cat# 100321, RRID:AB_389300
Armenian Hamster CD3e PE	Biolegend	Cat# 100308, RRID:AB_312673
Mouse NK1.1 PE	Biolegend	Cat# 108707, RRID:AB_313394
Rat CD4 PE-Cy7	Biolegend	Cat# 100422, RRID:AB_312707
Rat CD8a 780	Biolegend	Cat# 100714, RRID:AB_312753
Rat CD8a BV-785	Biolegend	Cat# 100749, RRID:AB_11218801
Rat CD8a PE	Biolegend	Cat# 100708, RRID:AB_312747
Rat FOXP3 PerCp Cy5.5	eBioscience	Cat# 45-5773-82, RRID:AB_914351
Rat Lag3 Biotin	Biolegend	Cat# 125205, RRID:AB_961177
Rat PDL1 PE-Cy7	Biolegend	Cat# 124314, RRID:AB_10643573
Rat PDL1 APC	Biolegend	Cat# 124312, RRID:AB_10612741
Rat Arginase 1 APC	eBioscience	Cat# 17-3697-82, RRID:AB_2734835
Rat PD1 APC	Biolegend	Cat# 109112, RRID:AB_10612938
Rat Ki67	Biolegend	Cat# 652418, RRID:AB_2564269
Rat IL-7Ra APC	Biolegend	Cat# 135011, RRID:AB_1937217
Rat B220 488	Biolegend	Cat# 103228, RRID:AB_492874
Rat CD11b APC-Cy7	Biolegend	Cat# 101226, RRID:AB_830642)
Rat CD11b 647	Biolegend	Cat# 101218, RRID:AB_389327
Armenian Hamster CD11c PE-Cy7	Biolegend	Cat# 117318, RRID:AB_493568
Rat Ly6C FITC	BD PharMingen	Cat# 561085, RRID:AB_10584332
Rat Ly6G PE-Cy7	Biolegend	Cat# 127617, RRID:AB_1877262
Rat F4/80 FITC	eBioscience	Cat# 11-4801-82, RRID:AB_2637191
Rat F4/80 APC-eFluor780	eBioscience	Cat# 47-4801-82, RRID:AB_2735036
Mouse aSMA 488	Thermo Fisher	Cat# 53-9760-82, RRID:AB_2574461
Mouse aSMA eFluor570	Thermo Fisher	Cat# 41-9760-82, RRID:AB_2573631)
Rat PDGFRa Biotin	Biolegend	Cat# 135910, RRID:AB_2043974
Rat PDGFRb Biotin	Biolegend	Cat# 136010, RRID:AB_2236916
Rat Thy1 APC-Cy7	Biolegend	Cat# 105328, RRID:AB_10613293
Armenian Hamster CD34 APC	Biolegend	Cat# 119310, RRID:AB_1236469
Rat CD34 660	eBioscience	Cat# 50-0341-82, RRID:AB_10596826
Mouse CXCL12 PE	R&D Systems	Cat# IC350C, RRID:AB_1964552
Rat C3 PE	Novus	Cat# NB200-540, RRID:AB_10003444
Syrian Hamster PDPN	Biolegend	Cat# 127402, RRID:AB_1089187
Rabbit αSMA	abcam	Cat# ab5694, RRID:AB_2223021
Goat PDGFRα	R&D Systems	Cat# AF1062, RRID:AB_2236897
Rat CD34	eBioscience	Cat# 14-0341-82, RRID:AB_467210
Rat CD34 488	eBioscience	Cat# 11-0341-82, RRID:AB_465021

(*Continued on next page*)

Continued

REAGENT or RESOURCE	SOURCE	IDENTIFIER
Rat F4/80	AbDserotech	Cat# MCA497, RRID:AB_2098196
Rat F4/80 488	AbDserotech	Cat# MCA497A488T, RRID:AB_1102554
Rat CXCR4	R & D Systems	Cat# MAB21651, RRID:AB_2261636
Sheep CSFR1	R & D Systems	Cat# AF3818, RRID:AB_884158
Rabbit CSF1	ABGENT	ABO12249
Rabbit NG2	abcam	Cat# ab83178, RRID:AB_10672215
Rat CD31	Biolegend	Cat# 102502, RRID:AB_312909
Rabbit C3aR	Invitrogen	Cat# PA5-29979, RRID:AB_2547453
Rat Ly6C APC	BD PharMingen	Cat# 560595, RRID:AB_1727554
Rat Cd11b biotin	Biolegend	Cat# 101204, RRID:AB_312787
Chemicals, Peptides, and Recombinant Proteins		
Anti C3a	Hycult Biotech	Cat# HM1072, RRID:AB_10130227
SB 290157	Sigma	cat# 559410
Deposited Data		
scRNA-seq	This paper	ArrayExpress: E-MTAB-7427
scRNA-seq	This paper	ArrayExpress: E-MTAB-7417
Experimental Models: Cell Lines		
B16.F10 melanoma cell line	American Type Culture Collection (ATCC)	ATCC Cat# CRL-6475, RRID:CVCL_0159
E0771 breast cancer cell line	CH3 BioSystems	Cat# 94A001 RRID:CVCL_GR23
Experimental Models: Organisms/Strains		
Mouse C57BL/6	Harlan	Reference# 057
Mouse C57BL/6-Tg(CAG-EGFP)131Osb/LeySopJ	Jackson Laboratory	Stock# 003291
Software and Algorithms		
Salmon (version 0.8.2)	(Patro et al., 2017)	https://combine-lab.github.io/salmon/
R version	R Foundation	https://www.r-project.org
Seurat (version 2.3.4)	(Satija et al., 2015)	https://satijalab.org/seurat/
Monocle (version 2.8.0)	(Qiu et al., 2017)	http://cole-trapnell-lab.github.io/monocle-release/
TraCeR	(Stubbington et al., 2016)	https://github.com/teichlab/tracer
gprofiler	(Raudvere et al., 2019)	https://biit.cs.ut.ee/gprofiler/gost
scran	(Scialdone et al., 2015)	https://bioconductor.org/packages/release/bioc/html/scran.html
CellPhoneDB	(Vento-Tormo et al., 2018)	https://www.cellphonedb.org/
CellRanger (version 2.2.0)	10x Genomics	https://www.10xgenomics.com/
Python	Python Software Foundation	https://www.python.org/
DESeq2	(Love et al., 2014)	https://bioconductor.org/packages/release/bioc/html/DESeq2.html
Other Datasets		
scRNA-seq	(Hagai et al., 2018)	ArrayExpress: E-MTAB-6831
Bulk RNA-seq	(Feig et al., 2013; Özdemir et al., 2015)	GEO: GSE42605
scRNA-seq	(Tirosh et al., 2016)	GEO: GSE72056
scRNA-seq	(Puram et al., 2017)	GEO: GSE103322
TCGA normalized expression data	TCGA (Colaprico et al., 2016)	R package TCGAbiolinks released under GPLv3 License http://bioconductor.org/packages/TCGAbiolinks/
Survival data	TCGA, ICGC (Cerami et al., 2012; Gao et al., 2013).	cBio Cancer Genomics Open Portal http://www.cbioportal.org

LEAD CONTACT AND MATERIALS AVAILABILITY

Further information and requests for resources and reagents should be directed to and will be fulfilled by the Lead Contact, Jacqueline D Shields (js970@mrc-cu.cam.ac.uk)

EXPERIMENTAL MODEL AND SUBJECT DETAILS

Cell lines

All cell lines were cultured according to protocols provided by the suppliers. The C57BL/6 derived B16.F10 melanoma cell line was purchased from American Type Culture Collection (ATCC) and cultured in Dulbecco's Modified Eagle medium (DMEM, Life Technologies), supplemented with 1% Penstrep and 10% FBS. 2.5 x10^5. The E0771 breast cancer cell line was purchased from CH3 BioSystems and cultured in RPMI (Sigma) supplemented with 10% FBS, 1% PS and 10mM HEPES. Cells were maintained at 37°C in 5% CO2 in a humidified incubator and passaged every 3 days.

Mice

Animals were housed in accordance with UK regulations and experiments were performed under project licenses PPL 80/2574 or PPL P8837835. Wild-type (WT) C57BL/6 mice, or C57BL/6-Tg(CAG-EGFP)131Osb/LeySopJ mice (Stock number 003291, Jackson Laboratory) were socially housed in individually ventilated cages with cage enrichment. Routine husbandry and care was performed by ARES facility staff in line with institutional guidelines. To generate chimeric mice, bone marrow was extracted from the femurs and tibias of CAG-EGFP mice. 20x10^5 bone marrow cells were injected intravenous (IV) into WT C57BL/6 irradiated mice (irradiated with 2x 5 Gray doses). Blood from chimeric mice was tested for bone marrow reconstitution before establishment of B16.F10 tumors. Sample sizes were calculated based on previous experience and *a priori* power analysis (G* Power). Animals recruited to studies remained socially housed in individually ventilated cages with cage enrichment and were not involved in prior regulated procedures. Animals were randomly assigned to experimental groups, and where possible, technicians performing the experiment were blinded to experimental groups and treatments.

Orthotopic syngeneic tumor models

B16 cells were subcutaneously injected into the shoulders of either 8 week old female immune competent wild-type (WT) C57BL/6 mice, or C57BL/6-Tg(CAG-EGFP)131Osb/LeySopJ mice (Jackson Laboratory). Animals were sacrificed and tissues collected for analysis at various time points. For breast tumors, 2.5 x10^5 E0771 cells were injected into the 4th inguinal mammary fat pad of 8 week old female C57BL/6 mice. Tumors were collected after 8 and 16 days of tumor development, whereas the fat pad itself was collected from non-tumor bearing mice.

Neutralising C3a/C3aR *in vivo*

B16.F10 shoulder allografts were allowed to develop for 5 days before treatment. Mice received 3 intraperitoneal (IP) injections of 10 μg/ml anti-C3a (HyCult Biotech, clone: 3/11) or IgG2a control (BioXCell, clone 2A3) 5, 7 and 9 days after tumor induction. Animals were randomized and technicians undertaking procedures were blinded to treatment groups. Animals were sacrificed 6 days (24hs after the first treatment) and 11 days post tumor induction and tumors isolated. Blood was collected by cardiac puncture and kept in capped EDTA tubes at RT before processing. Non-invasive tumor measurements were recorded daily and volumes calculated using the following formula $(\pi/6)(\text{shortest length*longest length})^2$. Data points from all animals were included unless tumors failed to form following technical issues with injection of cells. For C3aR antagonism, 100 μg (5% DMSO in PBS) of the small molecule SB290157 (Sigma) or vehicle control, was injected IP at either day 4 and 6 or day 8 and 10 into B16-F10 tumor bearing mice. Blood and tumor samples were collected 11 days post induction, as previously described.

EdU Incorporation

Tumor and non tumor bearing mice were injected IP with 200 μL pf 500 μg/ml 5-ethynyl-2′-deoxyuridine (EdU), at 48 and 24hs prior to culling. Skin form wt mice, as well as day 5 and day 11 tumors were collected for flow cytometry. Samples were processed and surface markers stained as previously described. Cells were fixed in 4% PFA (15mins) and permeabilised with saponin buffer (15mins). The Click-iT reaction was performed according to manufacturer's instructions, using the EdU Click-it Alexa Fluor 488 imaging kit (Invitrogen), before staining for intracellular targets was performed.

METHOD DETAILS

Tissue Processing

Tumors were mechanically dissociated and digested in 1mg/ml collagenase D (Roche), 1mg/ml collagenase A (Roche) and 0.4mg/ml DNase (Roche) in PBS, at 37°C for 2hs. Lymph nodes were mechanically dissociated and digested with 1mg/ml collagenase A (Roche) and 0.4mg/ml DNase (Roche) in PBS, at 37°C. After 30 mins, Collagenase D (Roche) was added (final concentration of 1mg/ml) to lymph node samples and digestion was continued for a further 30 mins. EDTA was added to all samples to neutralise

collagenase activity (final concentration (5mM) and digested tissues were passed through 70 μm filters (Flacon) ready for staining. 5ml of Red Blood Cell Lysis (RBC) lysis buffer (150mM NH_4Cl, 1mM $KHCO_3$, 0.1mM EDTA) was added to blood samples for 5 mins and neutralized with 45ml of PBS.

Isolation of Single Cells

Single cells were isolated from processed tissues using fluorescence-activated cell sorting (FACS). Once processed, samples were incubated with a fixable fluorescent viability stain (Life Technologies) for 20mins (diluted 1:1000 in PBS) prior to incubation with conjugated primary antibodies for 30 mins at 4°C. Antibodies were diluted 1:300 in PBS 0.5% BSA. Stained samples were index sorted, using the BD influx flow cytometer system, Single-cells were sorted into 2 μL of Lysis Buffer (1:20 solution of RNase Inhibitor (Clontech, cat. no. 2313A) in 0.2% v/v Triton X-100 (Sigma-Aldrich, cat. no. T9284)) in 96 well plates, spun down and immediately frozen at −80 degrees.

Preparation of cDNA and sequencing

Reverse transcription and cDNA pre-amplification were performed according to the SmartSeq2 protocol (Picelli et al., 2014) to obtain mRNA libraries from single-cells. Oligo-dT primer, dNTPs (ThermoFisher, cat. no. 10319879) and an ERCC RNA Spike-In Mix (1:50,000,000 final dilution, Ambion, cat. no. 4456740) were then added. Reverse Transcription and PCR were performed as previously published (Picelli et al., 2014), using 50U of SMARTScribe Reverse Transcriptase (Clontech, cat. no. 639538). cDNA libraries were prepared using the Nextera XT DNA Sample Preparation Kit (Illumina, cat. no. FC-131-1096), according to the protocol supplied by Fluidigm (PN 100-5950 B1). Single cell libraries were pooled, purified using AMPure XP beads (Beckman Coulter) and sequenced on an Illumina HiSeq 2500 aiming for an average depth of 1 Million reads/cell (paired-end 100-bp reads).

Flow Cytometry

Following a 20min incubation with a fixable fluorescent viability stain (see Isolation of Single Cells), cells were incubated with primary antibodies against cell surface markers, for 30mins at 4°C. Primary antibodies were diluted 1:300 in PBS 0.5% BSA according to STAR Methods. When analyzing immune cells, surface antibodies were diluted in a 50/50 mix of PBS 0.5% BSA and 2.4G2 FC Blocker (hybridoma supernatant generated in-house). If required, fluorescently labeled streptavidin, diluted 1:300 in PBS 0.5% BSA, was added for a further 30mins. To stain for intracellular targets, samples were fixed and permeabilized using the FOXP3 permeabilisation and fixation kit (eBioscence), for 1h at RT. Fixation and permeabilization was only performed once staining for surface markers was completed. Once samples were fixed, antibodies were diluted in a perm buffer from the FOXP3 permeabilisation and fixation kit, prepared according to manufacturer's instructions. Brefeldin-A (BFA, Biolegend) was used to investigate intracellular cytokine expression. BFA was added to the tissue digestion mix (1:1000) and samples were digested for 1h 30mins. After processing to a single cell suspension, samples were further incubated with BFA in media (1:1000 in DMEM 10% FBS) at 37°C for 2hs 30mins. Once staining was completed, samples were analyzed using the BD LSR-Fortessa system.

Immunofluorescence

Collected tissues were embedded in OCT medium (VWR) and snap frozen on dry ice. Frozen tissues were sectioned into 10 μm slices and stored at −80°C. Sections were air-dried and fixed in 50:50 acetone (Fluka): methanol (Fisher), at −20°C for 2mins or 4% paraformaldehyde (PFA) for 10 minutes at RT. If fixed with PFA, samples were permeabilized with 0.1% Triton for a further 10 minutes. After blocking for 1h at room temperature (RT) with blocking buffer (10% chicken serum and 2% Bovine Serum Albumin) in PBS, primary antibodies were applied overnight at 4°C or RT for 3hs. To visualize samples, secondary antibodies (Life Technologies), conjugated to either Alexa Fluor 488, 594 or 647, or fluorescently labeled streptavidin, were added for 2hs at RT. Samples were incubated with the nuclear stain 4',6-diamidino-2-phenylindole (DAPI) for 10 mins at 1 μg/ml, before mounting with ProLong Gold (ThermoFisher) liquid mountant. Streptavidin and secondary antibodies were diluted 1:300 in blocking buffer and primary antibodies (STAR Methods) were diluted in blocking buffer (for PDPN, CD31, CD11b biotin and F4/80, 1:100; for CD34 488, C3aR, F4/80 488 and Ly6C APC 1:20; for all other antibodies 1:50). Confocal imaging was performed using the Zeiss LSM 880 microscope and processed using the Zeiss Blue software. ROIs from 63x tile scans were included to show the presence of stromal components in sufficient detail.

QUANTIFICATION AND STATISTICAL ANALYSIS

Parameters such as sample size, measures of center, dispersion and precision (mean + SD or SEM) and statistical significance are reported in text, Figures and Figure Legends. Results were considered statistically significant when $p < 0.05$, by the appropriate test, as indicated in the text and Figure Legends.

Single-cell RNA sequencing analysis

The SmartSeq2 data was quantified with Salmon (Patro et al., 2017) (version 0.8.2), using the GENCODE mouse protein-coding transcript sequences. Transcript Per Million (TPM) values reported by Salmon were used for the quality control of the samples. In order to get the endogenous TPM values, we removed the ERCC's from the expression table and scaled the TPM's so that they sum to a

million. Cells with less than 1500 detected genes and for which the total mitochondrial expression exceeded 20% were excluded from further analysis. Genes that were expressed in less than 3 cells were also removed.

Downstream analysis, such as SNN graph-based clustering, differential expression analysis and visualization were performed using the Seurat package (Satija et al., 2015) (version 2.3.4) implemented in R. Clusters were identified using the community identification algorithm as implemented in the Seurat "FindClusters" function. The shared nearest neighbor graph was constructed using between 10 and 30 principal components as determined by the dataset variability; the resolution parameter to find the resulting number of clusters was tuned so that it produced a number of clusters large enough to capture most of the biological variability. Differential expression analysis was performed based on the Wilcoxon rank sum test. Clusters were annotated using canonical cell type markers. Two clusters of dDC2 in the tumor represented the same cell type and were therefore merged.

Trajectory modeling and pseudotemporal ordering of cells was performed with the Monocle 2 R-package (Qiu et al., 2017) (version 2.8.0). Briefly, the algorithm learns the sequence of expression changes each cell goes through as a part of a dynamics process and places each cell at its appropriate position in the trajectory. The most highly variable genes were used for ordering the cells. Potential doublets and contaminating melanocytes and keratinocytes were excluded. We also removed a cluster for which the top markers were genes associated with dissociation-induced effects. Genes which changed along the identified trajectory were identified by performing a likelihood ratio test using the function differentialGeneTest in the monocle 2 package.

To further identify subpopulations, we reanalysed the T cells, innate immune cells (myeloid and NK) and the CD31- stromal cells separately, using the same workflow as described above. To account for the cell cycle heterogeneity in the T cell subsets a cell cycle score was calculated for each cell and this score was then regressed out. We used the function "AddModuleScore" from Seurat and the list of G2M associated genes from Scialdone et al. to calculate a cell cycle score for each cell.

The gprofiler R package (Raudvere et al., 2019) was used to find enriched GO terms in KEGG Pathways. All significantly upregulated genes (gSCS adjusted p value < 0.05) for populations were tested, using moderate hierarchical filtering.

T cell receptor (TCR) analysis

The TCR sequences for each single T cell were assembled using TraCeR (Stubbington et al., 2016) which allowed the reconstruction of the TCRs from scRNA-seq data and their expression abundance (transcripts per million, TPM), as well as identification of the size, diversity and lineage relation of clonal subpopulations. In total, we detected 77 TCR sequences with at least one paired productive $\alpha\beta$ or gamma-delta chain. Cells for which more than two recombinants were identified were excluded from further analysis.

Cell cycle analysis

The pair-based prediction method described by Scialdone et al. (Scialdone et al., 2015). and implemented in the R package scran was used to assign each cell a cell cycle stage. Briefly, using training data, pairs of marker genes are identified such that the expression of the first gene in the training data is greater than the second in a certain cell cycle stage but less than the second in all other stages. For each cell then, the method calculates the proportion of all marker pairs where the expression of the first gene is greater than the second in the test data.

Putative interactions between cell types

To enable a systematic analysis of cell-cell communication, we used CellPhoneDB (Vento-Tormo et al., 2018). CellPhoneDB is a manual curated repository of ligands, receptors and their interactions, integrated with a statistical framework for inferring cell-cell communication networks from single cell transcriptome data. For the mouse dataset, we used the ortholog genes.

Briefly, in order to identify the most relevant interactions between cell types, we looked for the cell-type specific interactions between ligands and receptors. Only receptors and ligands expressed in more than 10% of the cells in the specific cluster were considered. We performed pairwise comparisons between all cell types. First, we randomly permuted the cluster labels of all cells 1000 times and determined the mean of the average receptor expression level of a cluster and the average ligand expression level of the interacting cluster. For each receptor-ligand pair in each pairwise comparison between two cell types, this generated a null distribution. By calculating the proportion of the means which are as or higher than the actual mean, we obtained a *p-value* for the likelihood of cell type-specificity of a given receptor-ligand complex. We then prioritized interactions that are highly enriched between cell types based on the number of significant pairs and manually selected biologically relevant ones. For the multi-subunit heteromeric complexes, we required that all subunits of the complex are expressed (using a threshold of 10%), and therefore we used the member of the complex with the minimum average expression to perform the random shuffling.

Mouse skin fibroblasts from healthy mice

Skin samples from two 8-week old female C57BL/6 mice were processed, first by mechanical processing, followed by a 2h incubation with 0.5% collagenase B (Roche; 11088815001). Cells were then counted and loaded on the 10x Chromium machine. Libraries were prepared following the Chromium Single Cell 3′ v2 Reagent Kit Manual (Zheng et al., 2017). Libraries were sequenced on an Illumina HiSeq 4000 instrument with 26 bp for read 1 and 98 bp for read 2. Droplet-based sequencing data was aligned, filtered and quantified using the Cell Ranger Single-Cell Software Suite (version 2.2.0), against the mouse reference genome provided by Cell Ranger. The data was analyzed using the pipeline described above. Only the clusters identified as fibroblasts (based on expression of Col1a1, Col1a2) were considered for comparison with the stromal clusters.

Human skin fibroblasts

scRNA-seq data was downloaded from ArrayExpress: E-MTAB-6831 (Hagai et al., 2018). CD45-negative cells from a digested skin sample were taken from a human female and processed in a 10X Chromium machine (10X Genomics). Droplet-based sequencing data was aligned, filtered and quantified using the Cell Ranger Single-Cell Software Suite (version 1.2.0), against the GRCh38 human reference genome provided by Cell Ranger. The data was analyzed using the pipeline described above. Only the clusters identified as fibroblasts (based on expression of COL1A1, COL1A2) were considered for comparison with the stromal clusters.

Comparison of human and mouse skin fibroblasts with stromal clusters

To compare the mouse and human skin fibroblasts with the tumor stromal populations, a logistic regression with L2-norm regularization and a multinomial learning approach as described in La Manno et al. (2016) (implemented by the scikit-learn function LogisticRegression) was trained on the stromal clusters, using the log-transformed normalized data. The model was used to predict the probabilities of each mouse and human skin cell belonging to each one of the stromal clusters (implemented by the predict_proba function).

Public data analysis

Genes that were differentially expressed between populations in the bulk RNA seq data of sorted fibroblasts from KPC tumors and normal pancreas were found using R package DESeq2 (Love et al., 2014). The processed human melanoma dataset (Tirosh et al., 2016) and human head and neck cancer dataset (Puram et al., 2017) was analyzed using the pipeline described above. Clusters were annotated using canonical cell type markers. Progression free survival analysis on the TCGA data was performed using the survival R package (Therneau and Grambsch, 2000) and the patients were dichtomised based on the median expression value of C3. Kaplan-Meier estimator of survival was used to construct the survival curves. Log rank tests were used to compare progression free survival between patients in different groups.

DATA AND CODE AVAILABILITY

The raw sequencing data for the melanoma model has been deposited in ArrayExpress: E-MTAB-7427 and the count table can be downloaded from https://www.ebi.ac.uk/gxa/sc/experiments/E-EHCA-2/Results. We have also made this data available for online browsing with a user-friendly interface at http://www.teichlab.org/data/. The mouse skin data from healthy mice was deposited in ArrayExpress: E-MTAB-7417. Other data is available from the corresponding author on reasonable request. The human skin fibroblast data was downloaded from ArrayExpress: E-MTAB-6831(Hagai et al., 2018). Bulk RNA seq data of sorted fibroblasts from KPC tumors and normal pancreas was downloaded from GEO: GSE42605. The processed human melanoma dataset (Tirosh et al., 2016) and human head and neck cancer dataset (Puram et al., 2017) were downloaded from GEO: GSE72056, and GEO: GSE103322. The TCGA normalized expression data was downloaded using the R package TCGAbiolinks (Colaprico et al., 2016). The clinical data was downloaded from the cBioPortal (Cerami et al., 2012; Gao et al., 2013).

Cell Reports, Volume *31*

Supplemental Information

Single-Cell RNA Sequencing Reveals a Dynamic Stromal Niche That Supports Tumor Growth

Sarah Davidson, Mirjana Efremova, Angela Riedel, Bidesh Mahata, Jhuma Pramanik, Jani Huuhtanen, Gozde Kar, Roser Vento-Tormo, Tzachi Hagai, Xi Chen, Muzlifah A. Haniffa, Jacqueline D. Shields, and Sarah A. Teichmann

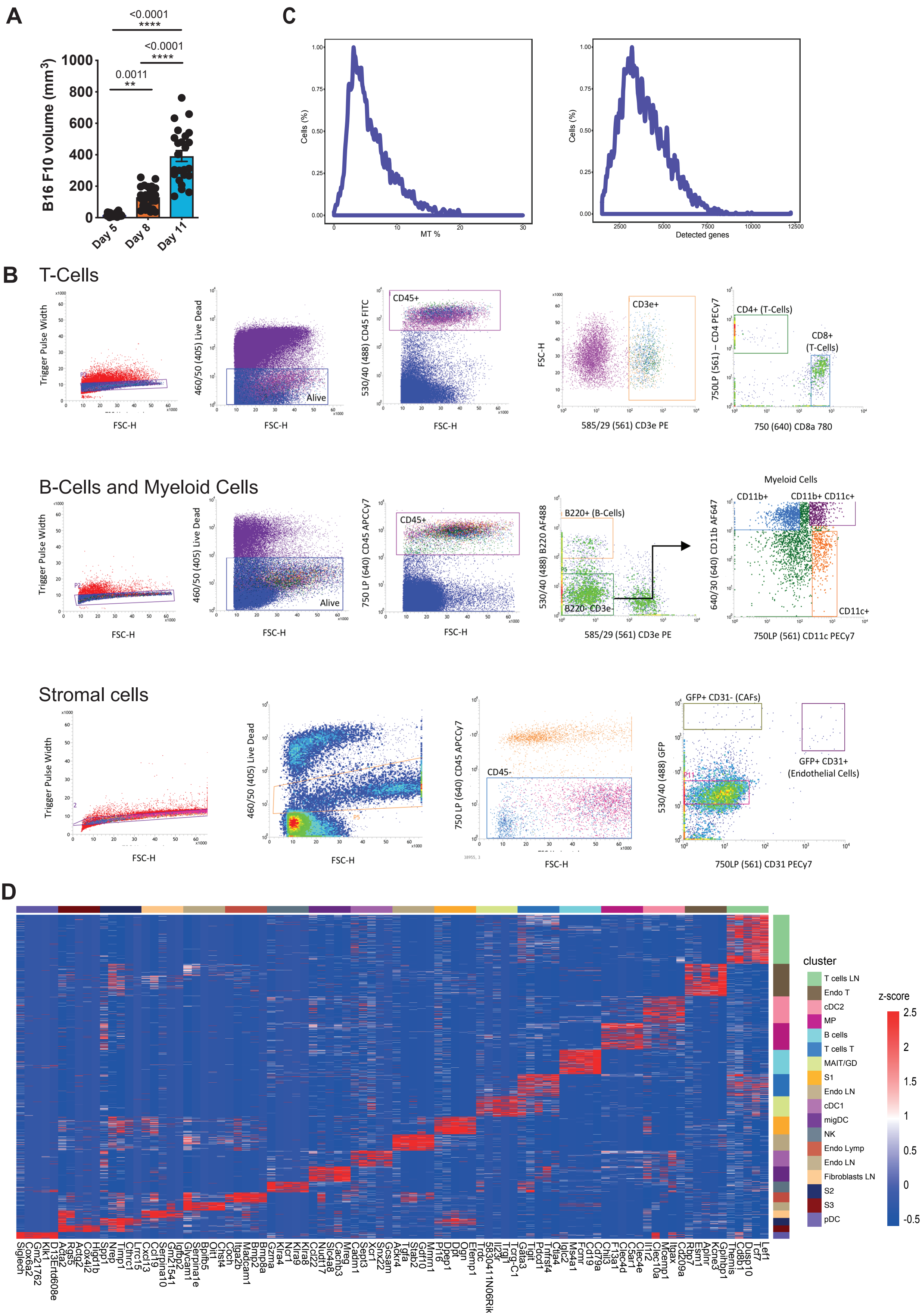

Supplementary Figure 1. Gating strategy and quality control of SS2 data, Related to Figure

1. (A) Volumes of B16-F10 melanomas at day 5, day 8 and day 11. Data presented as mean ± SEM * $P<0.05$, ** $P<0.01$, *** $P<0.001$, **** $P<0.0001$, one way Anova with Tukey post-hoc test, n = 22 individual mice. **(C)** Gating strategy for index sorted cell populations. **(B)** Quality control of the scRNA-seq dataset. Histograms show the distribution of the cells that passed the computational quality control ordered by number of detected genes and mitochondrial gene expression content. **(D)** Heatmap of marker genes showing relative expression (z-score) of the top 5 markers for each cluster presented in Fig. 1B.

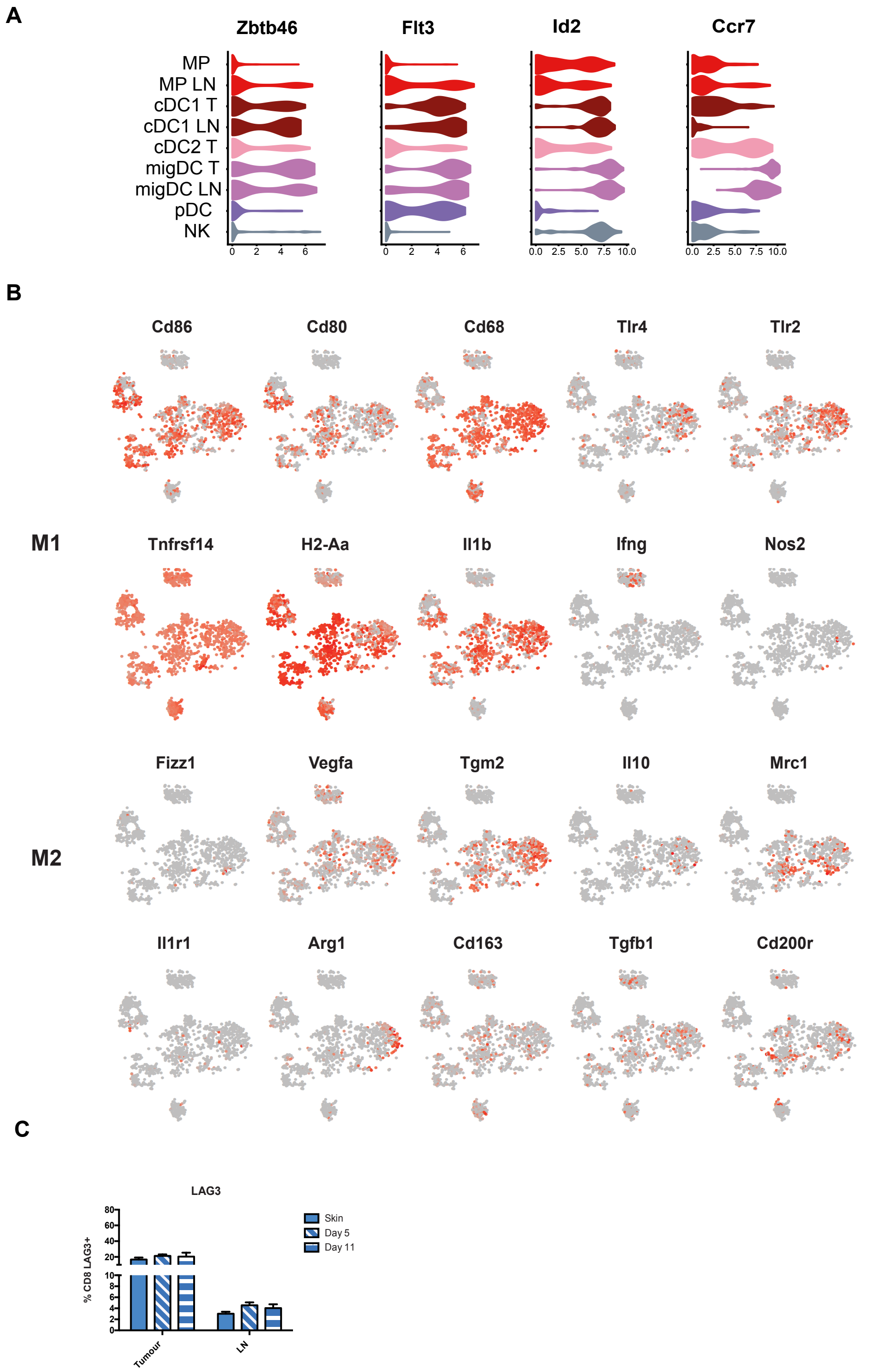

Supplementary Figure 2. Immune phenotypes, Related to Figures 2 and 3. (A) Violin plots showing expression of cDC1 transcription factors for each innate immune subpopulation. **(B)** tSNE plots showing expression of M1 and M2 macrophage markers in the innate immune subpopulations. Red indicates high expression, grey indicates low expression. **(C)** Percentage

of Lag3$^+$ (displayed as a percentage of total CD8$^+$ T cells), in tumours and lymph nodes isolated from skin, day 5 and day 11 tumour bearing or control mice. Data presented as mean ± SEM. n=4 independent mice per time point.

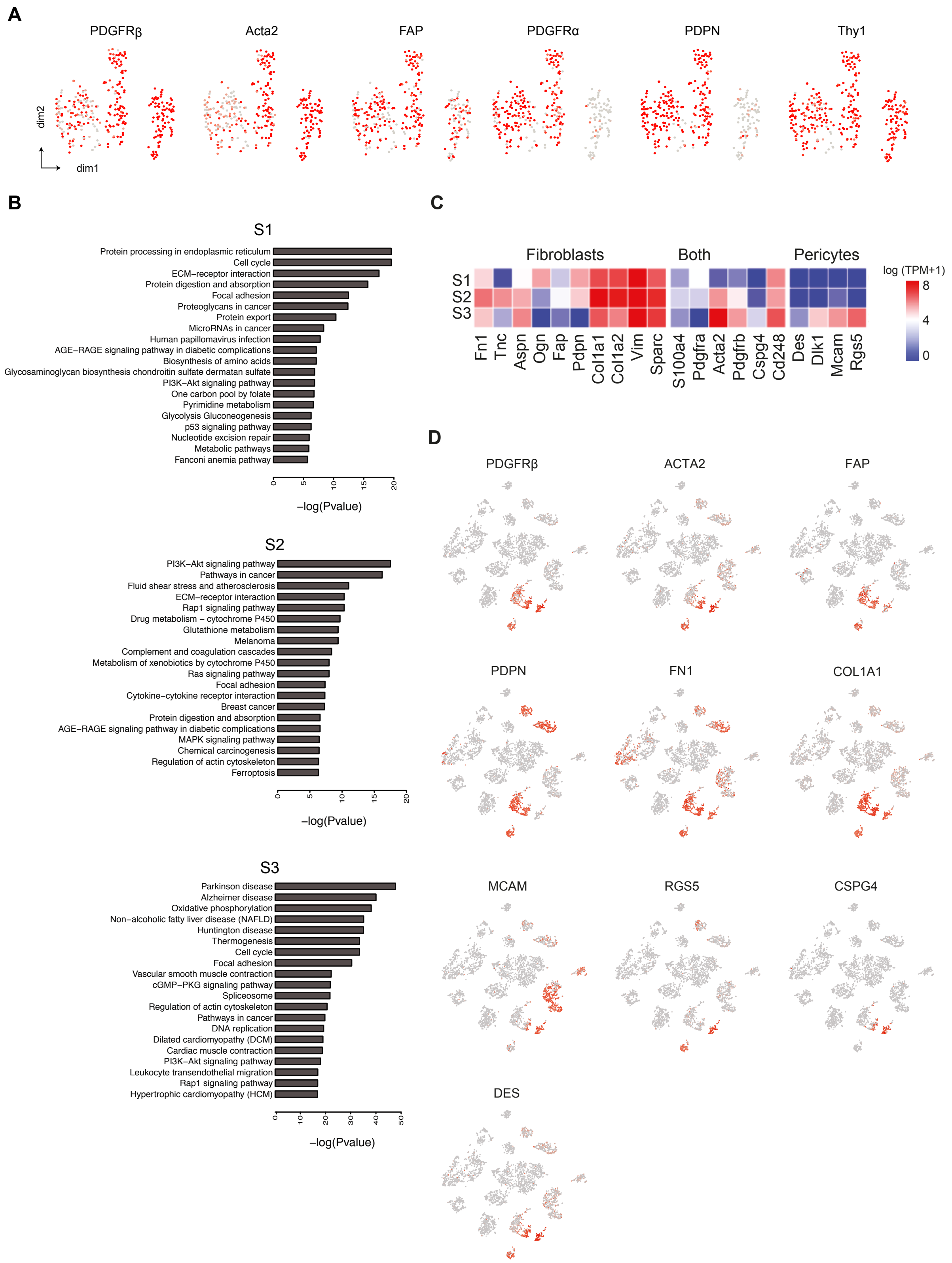

Supplementary Figure 3. Distinct stromal subpopulations identified in the melanoma mouse model, Related to Figure 4. (A) tSNE plots showing expression of typical CAF markers. **(B)** Bar plot (-log pvalue) depicting the top 20 gene ontology pathways upregulated in each stromal population. **(C)** Heatmap showing expression of canonical fibroblasts and pericytes

markers in the three stromal populations. **(D)** tSNE plots of all sequenced cells, showing expression of typical pericyte markers is also detected in $PDPN^+$ lymph node fibroblasts.

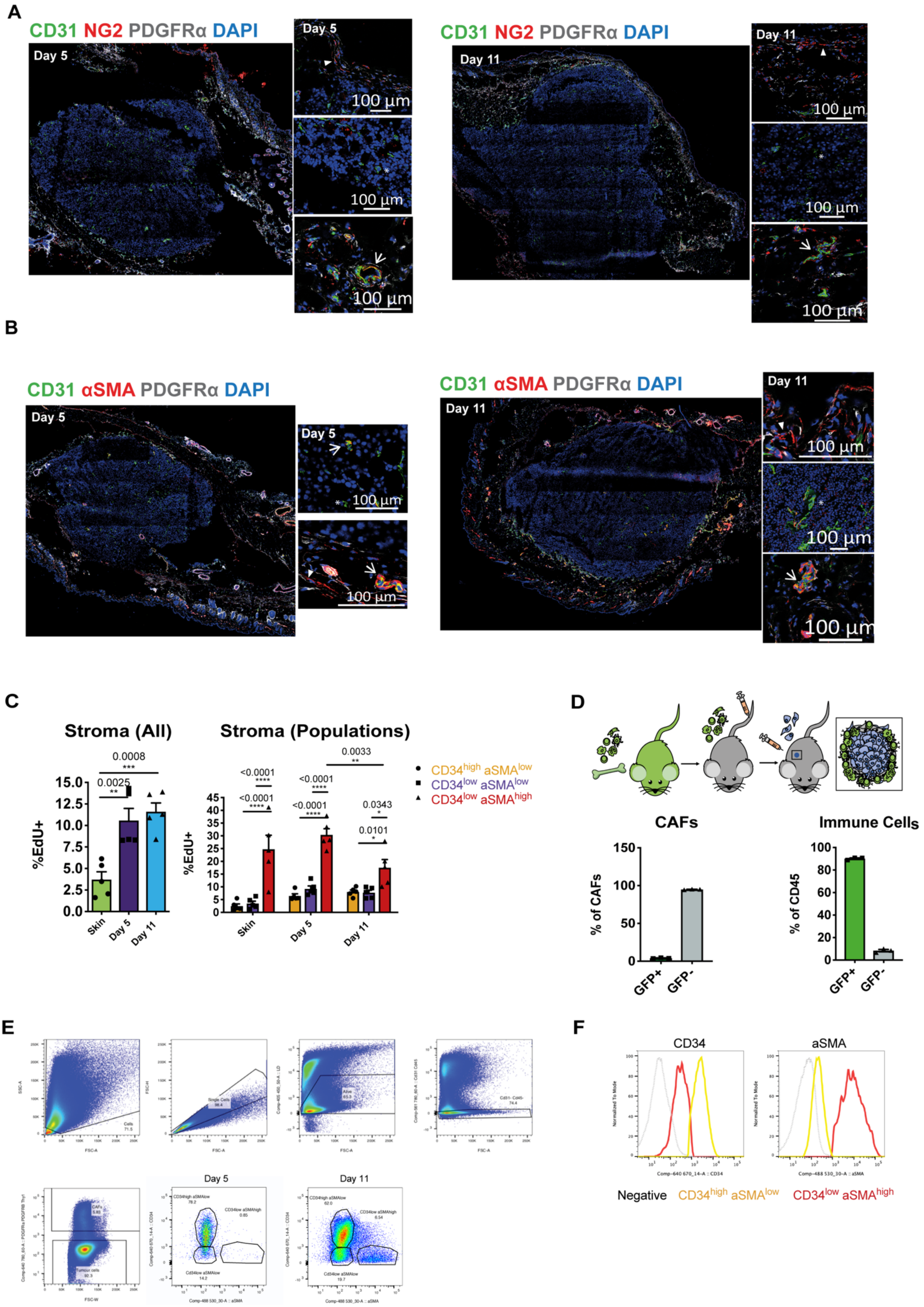
A
CD31 NG2 PDGFRα DAPI
Day 5
100 µm
Day 11
B
CD31 αSMA PDGFRα DAPI
C
Stroma (All)
%EdU+
0.0008
***
0.0025
**
Skin
Day 5
Day 11
Stroma (Populations)
<0.0001
****
0.0033
**
0.0343
*
0.0101
*
CD34high aSMAlow
CD34low aSMAlow
CD34low aSMAhigh
D
CAFs
% of CAFs
GFP+
GFP-
Immune Cells
% of CD45
E
Day 5
Day 11
F
CD34
aSMA
Negative
CD34high aSMAlow
CD34low aSMAhigh

Supplementary Figure 4. Validation of CAF markers and CAF origin, Related to Figures 4 and 5. (A) and **(B)** Confocal imaging showing αSMA$^+$ and NG2$^+$ cells both distinct from and associated with CD31$^+$ blood vessels. 20x tile scans of whole tumours are displayed, as well as 63x regions of interest. **(C)** EdU incorporation in stromal populations in skin, day 5 and day 11 tumours (left: % of CD45- CD31- Thy1+, right; individual subsets (S1-S3) incorporation into all proliferating fibroblasts (top) and each subset (bottom) in skin, day 5 and day 11 tumours. * $P<0.05$, ** $P<0.01$, *** $P<0.001$, **** $P<0.0001$ One way (left) or two way (right) ANOVA with a Tukey post-hoc test. **(D)** Diagram (top) depicting the generation of bone marrow chimeric mice by injecting (IV) bone marrow from CAG-EGFP mice into irradiated WT strains. B16-F10 tumours were injected into chimeric mice and allowed to form for 11 days. The proportion of bone derived marrow cells (GFP+) to stromal (CD45- CD31- Thy1+, bottom left) and immune (CD45+, bottom right) populations was assessed by flow cytometry. **(E)** Gating strategy used to identify stromal subsets along tumour development, **(F)** showing representative plots from day 5 and 11 tumours and histograms of CD34 and aSMA levels between populations. Scale bars 100 µm

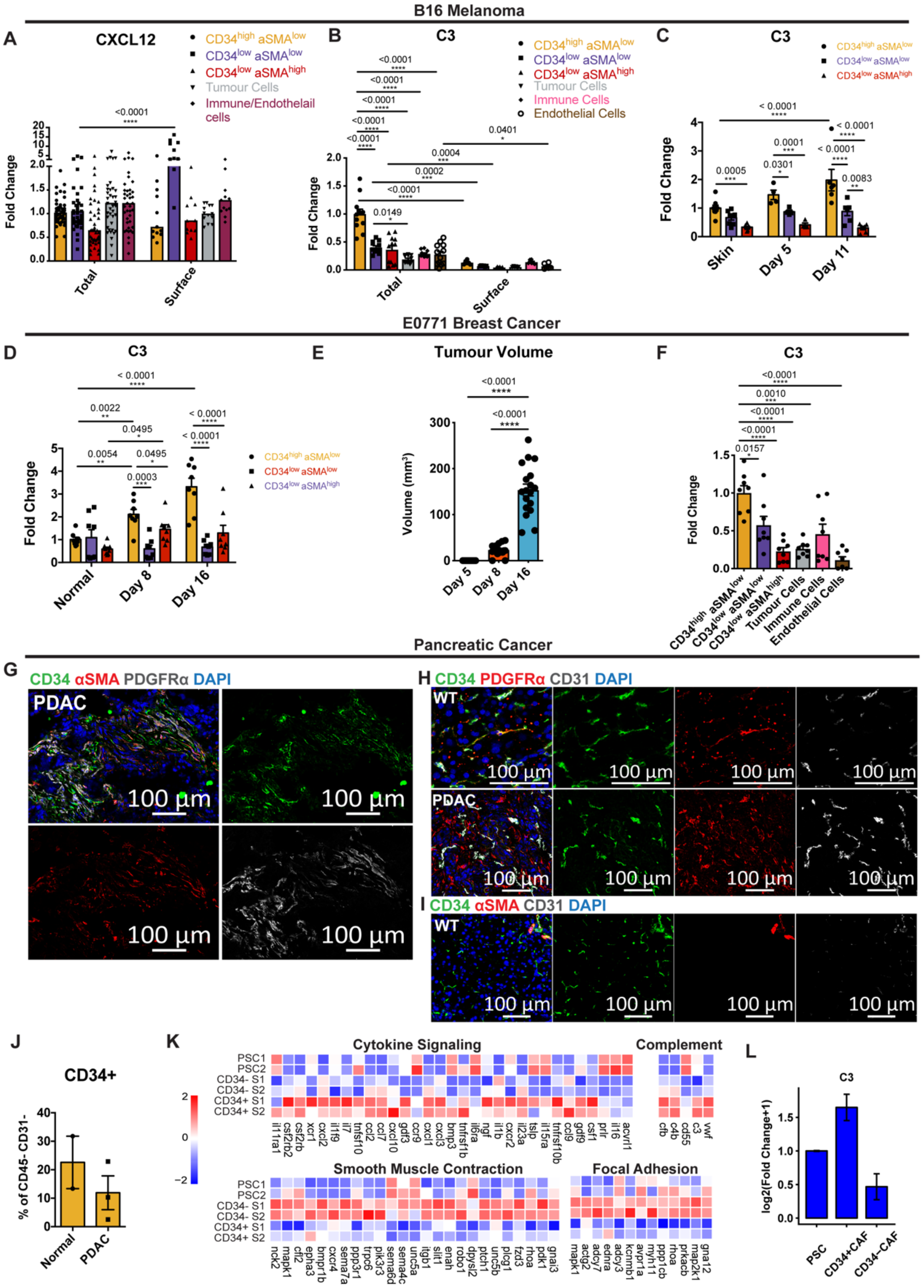

Supplementary Figure 5. Validation of stromal populations in mouse models of melanoma, breast and pancreatic cancer, Related to Figures 5 and 6. B16-F10 melanoma: Total and surface CXCL12 **(A)** and C3 **(B)** expression in day 11 tumours in stromal populations, immune, endothelial and tumour cells. CXCL12: n = minimum of 12 mice, C3: n = 6 mice. **(C)** C3 expression within each stromal population in normal skin, day 5 and day 11 tumours n =

minimum 4 mice. E0771 breast tumours: **(D)** C3 expression within fibroblast subsets in normal breast tissue, day 8 and day 16 tumours, n = 8 mice. **(E)** Tumour volumes at day 5, day 8 and day 16, n = 18 mice. **(F)** C3 expression in fibroblast populations, immune, endothelial and tumour cells in day 16 tumours, n = 8 mice. Pancreatic adenocarcinoma: **(G)** IF images of KPC tumours showing expression of CD34 (green), αSMA (red) and PDGFRα (grey). **(H)** IF images of WT pancreas compared to KPC tumours (PDAC), showing expression of CD34 (green), PDGFRα (red) and CD31 (grey). Pancreatic stellate cells in normal tissue express CD34 and PDGFRα, whereas CD34 expression is mostly restricted to CD31+ blood vessels in PDAC tissue. **(I)** IF images showing CD34 (green), αSMA (red) and CD31 (grey) in normal pancreatic tissue. Here, pancreatic stellate cells do not express αSMA. **(J)** Graph displays FACs quantification of CD34+ fibroblasts as a percentage of CD45-CD31- cells in normal pancreatic tissue and KPC tumours. normal pancreas: n = 2 mice, KPC tumours: n = 3 mice. **(K)** Publicly available RNA seq data of KPC CD34+ and CD34- stromal cells, as well as normal pancreatic stellate cells (PSCs) was downloaded and analysed. Heat maps show selected pathways from GO analysis performed on differentially expressed genes between CD34+ and CD34- populations, z scores are displayed. **(L)** Graph displaying log2(Fold change+1) of C3 RNA expression from PSCs, CD34+ and CD34- stromal cells. Graphs were made from previously mentioned publicly available data. ** $P<0.01$, *** $P<0.001$, **** $P<0.0001$, two way anova with a Sidak (A and B) and Tukey (C and D) post-hoc test, one way anova with Tukey post-hoc test. (E and F). All images are representative of at least n=3 mice and acquired at 63x, scale bars displayed.

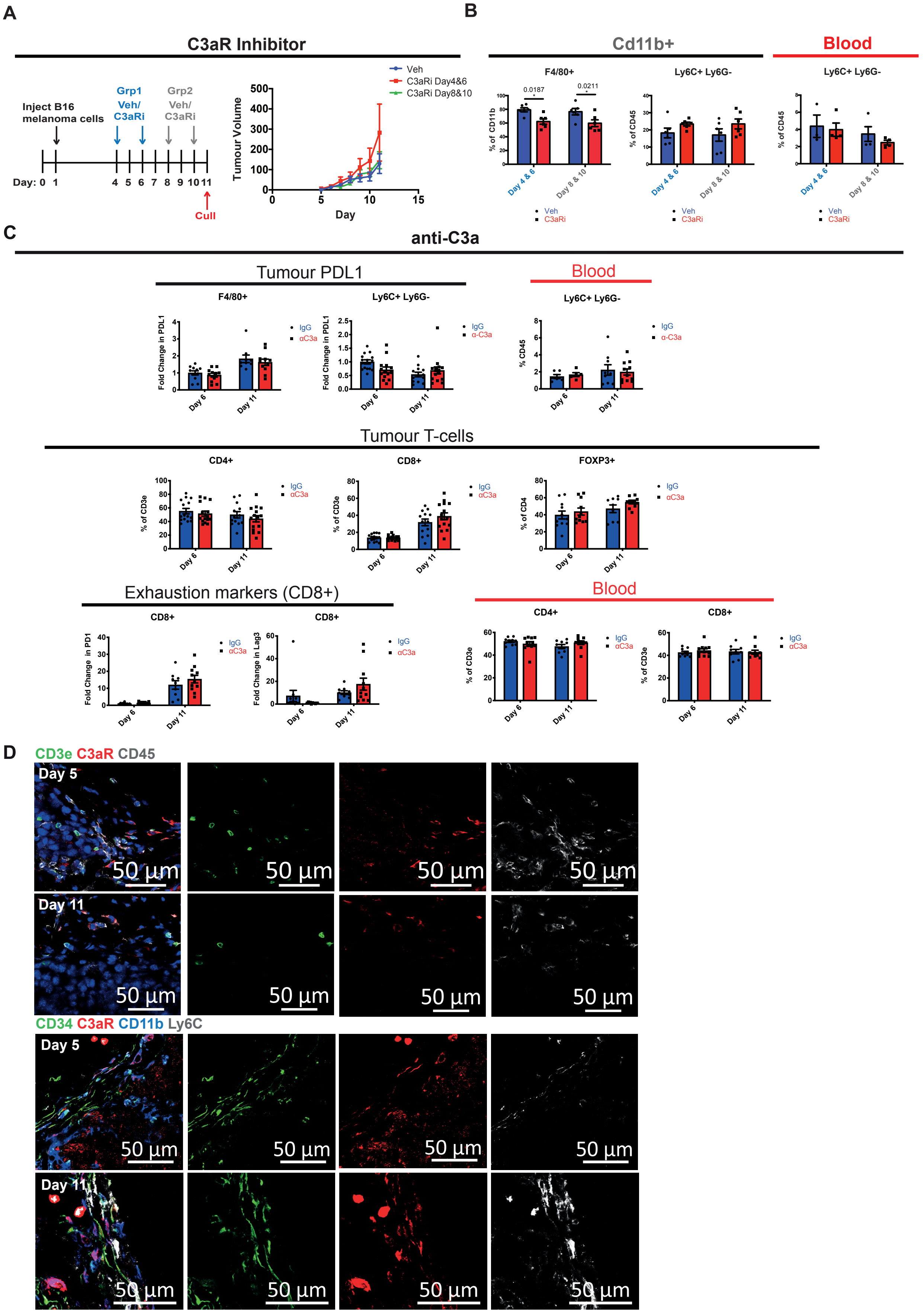
A
C3aR Inhibitor
Inject B16 melanoma cells
Grp1 Veh/ C3aRi
Grp2 Veh/ C3aRi
Day: 0 1 4 5 6 7 8 9 10 11
Cull
Tumour Volume
Day
Veh
C3aRi Day4&6
C3aRi Day8&10
B
Cd11b+
Blood
F4/80+
Ly6C+ Ly6G-
0.0187
0.0211
% of CD11b
% of CD45
Day 4 & 6
Day 8 & 10
Veh
C3aRi
C
anti-C3a
Tumour PDL1
Blood
Fold Change in PDL1
IgG
αC3a
α-C3a
Day 6
Day 11
Tumour T-cells
CD4+
CD8+
FOXP3+
% of CD3e
% of CD4
Exhaustion markers (CD8+)
Fold Change in PD1
Fold Change in Lag3
D
CD3e C3aR CD45
Day 5
Day 11
50 µm
CD34 C3aR CD11b Ly6C

Supplementary Figure 6. The effects of disrupting C3a/C3aR Interactions on tumour development and immune infiltration, Related to Figure 6. (A) Experimental design of C3aR antagonism, using the small molecule SB290157: Mice received injections of SB290157 at either day 4 and day 6 or day 8 and day 11, all mice were culled at day 11 (left). Tumour volumes (mm^3) for vehicle controls and each treatment regime are shown (right), n = 6 mice. **(B)** Immune infiltration of F4/80+ macrophages and Ly6C+ Ly6G- monocytes in the tumour (left) and blood (right). This is shown as the percentage of CD11b+ cells (F4/80) or CD45+ cells (Ly6C+ Ly6G-), n = 6 tumours. * = $P< 0.05$ (Two way anova with Sidak post-hoc test). **(C)** The effects of neutralising C3a on immune infiltrates in the tumour and blood. Top panel: Fold change in fluorescence intensity of PDL1 on tumour myeloid populations, normalized to IgG day6 (left) and the number of Ly6C+ Ly6G- monocytes in the blood, shown as a percentage of CD45 cells. Middle panel: Number of tumour infiltrating CD8+, CD4+ and FOXP3+ T-cells, displayed as a percentage of CD3e or CD4 T-cells. Bottom panel: Fold change in fluorescence intensity of T-cell exhaustion markers PD1 and Lag3 in CD8 T cells, normalized to IgG day 6 (left). Numbers of CD8 and CD4 T cells in the blood of treated mice, displayed as a percentage of CD3e T-cells. n = minimum of 11 mice. **(D)** Confocal images showing expression of C3aR on immune populations in day 5 and 11 tumours. Top panel: C3aR is not expressed on Cd3e+ T cells, CD3e (green), C3aR (red) and CD45 (grey). Bottom panel: C3aR is expressed by Ly6C+ CD11b+ monocytes. Images were acquired at 63x and scale bars are displayed. day 5 n=3 tumours, day 11 n=2 tumours.

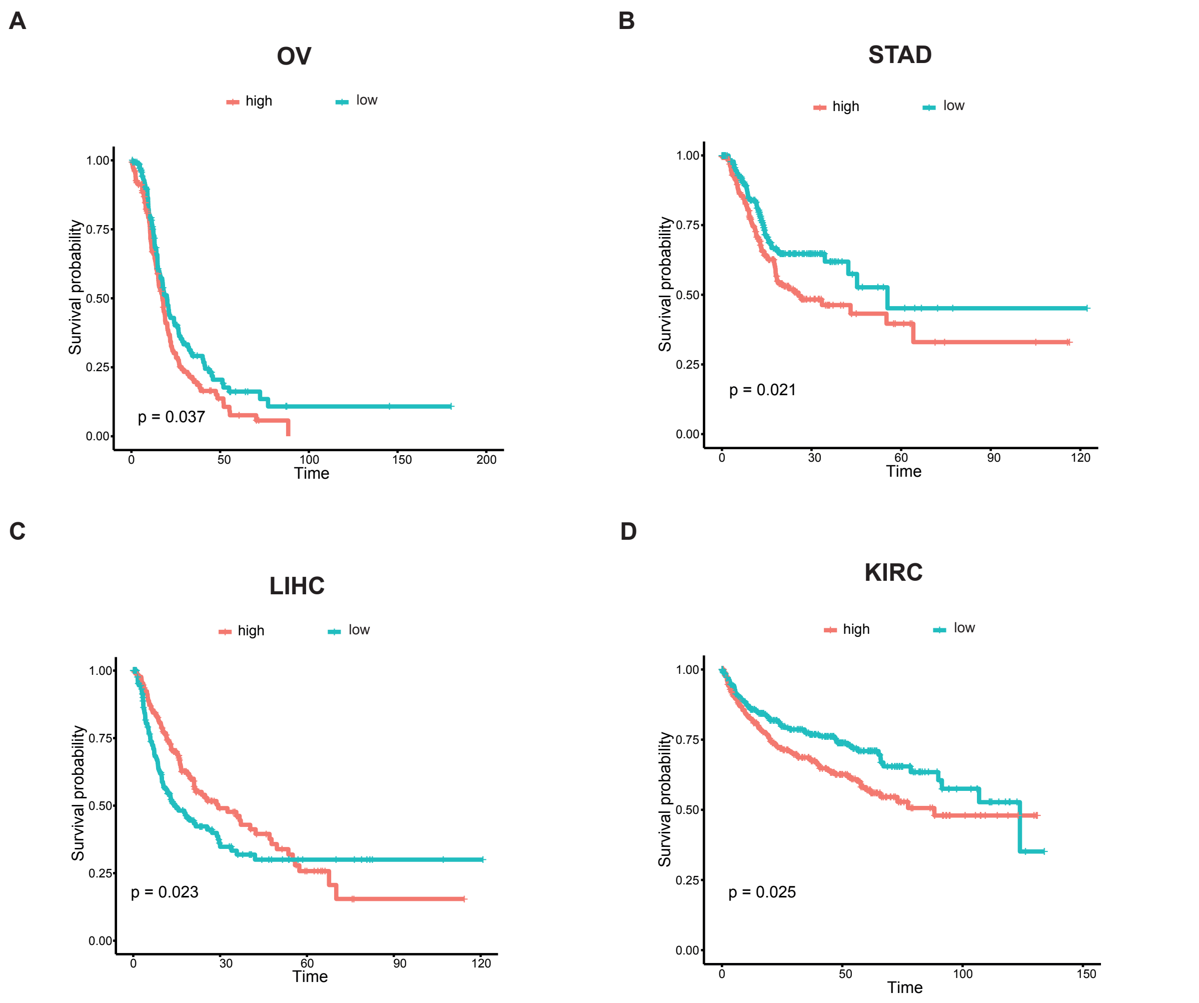

Supplementary Figure 7. Survival analysis of C3 in different TCGA datasets, Related to Figure 7. (A) Kaplan-Meier survival curve of Ovarian serous cystadenocarcinoma (OV) patients showing progression-free survival based on median C3 expression levels. **(B)** Kaplan-Meier survival curve of Stomach adenocarcinoma (STAD) patients showing progression-free survival based on median C3 expression levels. **(C)** Kaplan-Meier survival curve of Liver hepatocellular carcinoma (LIHC) patients showing progression-free survival based on median C3 expression levels. **(D)** Kaplan-Meier survival curve of Kidney renal clear cell carcinoma (KIRC) patients showing progression-free survival based on median C3 expression levels.
